# AI-powered prostate cancer detection: a multi-centre, multi-scanner validation study

**DOI:** 10.1007/s00330-024-11323-0

**Published:** 2025-02-28

**Authors:** Francesco Giganti, Nadia Moreira da Silva, Michael Yeung, Lucy Davies, Amy Frary, Mirjana Ferrer Rodriguez, Nikita Sushentsev, Nicholas Ashley, Adrian Andreou, Alison Bradley, Chris Wilson, Giles Maskell, Giorgio Brembilla, Iztok Caglic, Jakub Suchánek, Jobie Budd, Zobair Arya, Jonathan Aning, John Hayes, Mark De Bono, Nikhil Vasdev, Nimalan Sanmugalingam, Paul Burn, Raj Persad, Ramona Woitek, Richard Hindley, Sidath Liyanage, Sophie Squire, Tristan Barrett, Steffi Barwick, Mark Hinton, Anwar R. Padhani, Antony Rix, Aarti Shah, Evis Sala

**Affiliations:** 1 Department of Radiology, University College London Hospitals NHS Foundation Trust, London, UK; 2 Division of Surgery & Interventional Science, University College London, London, UK; 3 Lucida Medical Ltd, Cambridge, UK; 4 Cambridge University Hospitals NHS Foundation Trust & University of Cambridge, Cambridge, UK; 5 Royal Cornwall Hospitals NHS Trust, Truro, UK; 6 Royal United Hospitals Bath NHS Foundation Trust, Bath, UK; 7 IRCCS San Raffaele Scientific Institute, Vita-Salute San Raffaele University, Milan, Italy; 8 Fabrica AI Corp., Delaware City, DE USA; 9 North Bristol NHS Trust, Bristol, UK; 10 East and North Herts NHS Trust, Stevenage, UK; 11 University of Hertfordshire, Hatfield, UK; 12 Mid and South Essex NHS Foundation Trust, Southend, UK; 13 Somerset NHS Foundation Trust, Taunton, UK; 14 Research Center for Medical Image Analysis and Artificial Intelligence (MIAAI), Danube Private University, Krems an der Donau, Austria; 15 University of Winchester, Winchester, UK; 16 Hampshire Hospitals NHS Foundation Trust, Winchester, UK; 17 Paul Strickland Scanner Centre, Mount Vernon Hospital, Northwood, UK; 18 Dipartimento Diagnostica per Immagini e Radioterapia Oncologica, Policlinico Universitario A. Gemelli IRCCS, Rome, Italy; 19 Dipartimento di Scienze Radiologiche ed Ematologiche, Università Cattolica del Sacro Cuore, Rome, Italy

**Keywords:** Prostatic neoplasms, Magnetic resonance imaging, Artificial intelligence

## Abstract

**Objectives:**

Multi-centre, multi-vendor validation of artificial intelligence (AI) software to detect clinically significant prostate cancer (PCa) using multiparametric magnetic resonance imaging (MRI) is lacking. We compared a new AI solution, validated on a separate dataset from different UK hospitals, to the original multidisciplinary team (MDT)-supported radiologist’s interpretations.

**Materials and methods:**

A Conformité Européenne (CE)-marked deep-learning (DL) computer-aided detection (CAD) medical device (Pi) was trained to detect Gleason Grade Group (GG) ≥ 2 cancer using retrospective data from the PROSTATEx dataset and five UK hospitals (793 patients). Our separate validation dataset was on six machines from two manufacturers across six sites (252 patients). Data included in the study were from MRI scans performed between August 2018 to October 2022. Patients with a negative MRI who did not undergo biopsy were assumed to be negative (90.4% had prostate-specific antigen density < 0.15 ng/mL^2^). ROC analysis was used to compare radiologists who used a 5-category suspicion score.

**Results:**

GG ≥ 2 prevalence in the validation set was 31%. Evaluated per patient, Pi was non-inferior to radiologists (considering a 10% performance difference as acceptable), with an area under the curve (AUC) of 0.91 vs. 0.95. At the predetermined risk threshold of 3.5, the AI software’s sensitivity was 95% and specificity 67%, while radiologists at Prostate Imaging-Reporting and Data Systems/Likert ≥ 3 identified GG ≥ 2 with a sensitivity of 99% and specificity of 73%. AI performed well per-site (AUC ≥ 0.83) at the patient-level independent of scanner age and field strength.

**Conclusion:**

Real-world data testing suggests that Pi matches the performance of MDT-supported radiologists in GG ≥ 2 PCa detection and generalises to multiple sites, scanner vendors, and models.

**Key Points:**

***Question*** *The performance of artificial intelligence-based medical tools for prostate MRI has yet to be evaluated on multi-centre, multi-vendor data to assess generalisability.*

***Findings*** *A dedicated AI medical tool matches the performance of multidisciplinary team-supported radiologists in prostate cancer detection and generalises to multiple sites and scanners.*

***Clinical relevance*** *This software has the potential to support the MRI process for biopsy decision-making and target identification, but future prospective studies, where lesions identified by artificial intelligence are biopsied separately, are needed.*

**Graphical Abstract:**

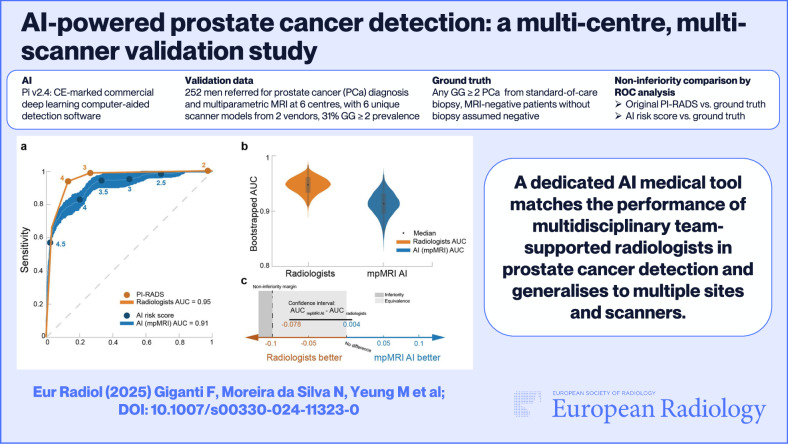

## Introduction

Magnetic resonance imaging (MRI) is a valuable tool for pre-biopsy assessment and early detection of prostate cancer (PCa), as it significantly improves patient outcomes by allowing for timely intervention and more effective treatment options [1, 2].

Progress has been made with the development of the Prostate Imaging-Reporting and Data Systems (PI-RADS) scoring system [3]. However, variability in cancer detection performance across centres is influenced by several key factors. Differences in radiologist training, along with variations in scanner technology and imaging protocols, play a significant role in the consistency and accuracy of cancer detection outcomes [4].

There is growing interest in applying deep-learning-based computer-aided detection (DL-CAD) software to improve the detection accuracy for clinically significant PCa (csPCa), usually defined as Grade Group (GG) ≥ 2 [5, 6]. DL-CAD systems for PCa detection have performance approaching that of expert radiologists [7]. By assisting radiologists with more precise image analysis, these systems may also help reduce the need for invasive biopsies, ultimately enhancing patient care. However, studies have largely remained limited to a retrospective, single-site, single-scanner data, preventing assessments of model generalisation. In the systematic review by Sushentsev et al [8], which compared fully automated and semi-automated MRI-based AI algorithms for differentiating csPCa, 17 studies passed the quality screening. Of these, 14 (82%) were conducted at a single site and used data from a single vendor. For translation into clinical practice, performance evaluation on multi-centre, multi-vendor data is crucial to assess generalisability.

We compared a proprietary Conformité Européenne (CE)-certified DL-CAD medical device (Lucida Medical, Prostate Intelligence™-Pi-v2.4), intended to provide risk scores for the likelihood of csPCa, to the original multidisciplinary team (MDT)-supported radiologist’s interpretations with a separate validation dataset from different UK hospitals.

## Methods

### Data source and study population

The study sponsor was Hampshire Hospitals NHS Foundation Trust and received ethical approval and waiver of consent from the UK HRA (IRAS #278640). Lucida Medical Ltd provided funding for the study. Data were collected from six hospitals in the UK National Health Service with varying scanner models and acquisition protocols through a retrospective, multi-centre, cohort study (PAIR-1). Patients included were referred between 2018 to 2022 for MRI and underwent biopsy according to the local standard of care.

Inclusion criteria were: (i) patients ≥ 21 years old referred for prostate MRI for suspected PCa; (ii) concordance between the original MRI report (PI-RADS or Likert score) done by expert genitourinary radiologists (i.e., > 1000 prostate MR cases reported) [9] and biopsy, either performed following MRI or not recommended due to negative MRI.

Excluded patients were those: (i) not scanned with the centre’s preferred MRI scanner or protocol; (ii) who declined a recommended biopsy or had missing biopsy results; (iii) with poor-quality scans; (iv) prior PCa diagnosis.

**Fig. 1:**
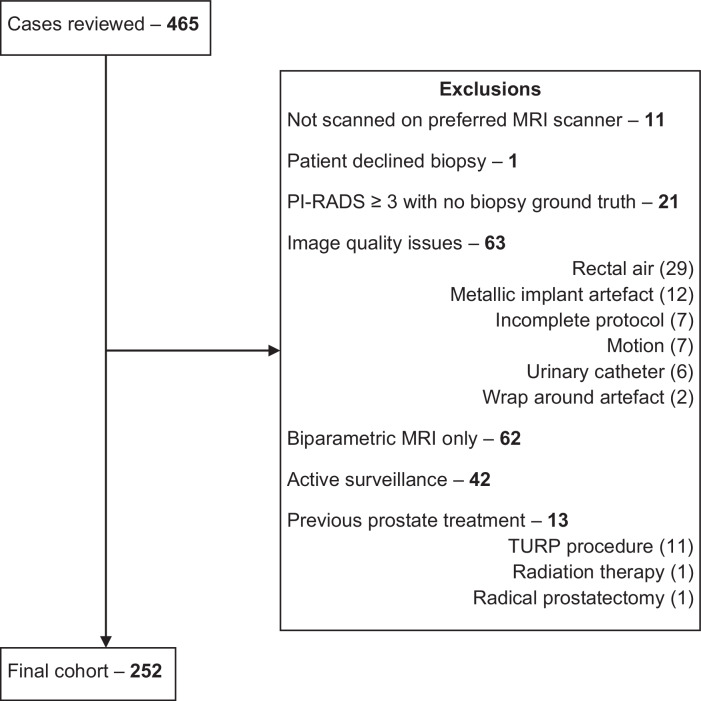
CONSORT diagram illustrating each step for patient selection in the validation and reasons for exclusions. PI-RADS, Prostate Imaging-Reporting And Data System; MRI, magnetic resonance imaging; TURP, transurethral resection of the prostate

The CONSORT diagram for the validation set is in Fig. 1.

### Ground truth

Histopathology results from biopsies were used to confirm GG ≥ 2 cancers. All centres conducted transperineal biopsies, using either cognitive or fusion techniques. Targeted + systematic biopsy was performed according to local standard-of-care after MDT discussions.

Lesions indicating likely GG ≥ 2 cancers were manually marked on MRI images by one of three expert radiologists using histopathology reports [9]. MRI-visible lesions were noted even when originally unreported. All annotations on the validation set were independently verified by another expert genitourinary radiologist [9]. Where biopsy was not obtained, and csPCa was not suspected in the original MRI report, cases were assumed negative (90.4% of these patients not at elevated risk of GG ≥ 2 by the prostate-specific antigen (PSA) density (PSAD) (< 0.15 ng mL^2^) [1]. More details on the reading strategy followed in this study are available in Supplementary Material S1.

### Development and evaluation data

AI development used the earliest 70% of cases that met all the eligibility criteria from five PAIR-1 sites to minimise data leakage compared to the later acquired validation data (*n* = 841). The publicly available PROSTATEx dataset was also used for the artificial intelligence (AI) model development (*n* = 204) [10–12]. The PROSTATEx dataset comprises multiparametric MRI (mpMRI) (T2-weighted, diffusion-weighted imaging, and dynamic contrast-enhanced imaging) scans from 204 patients, acquired on two Siemens 3-T scanner (Magnetom Trio and Skyra). The mean age of patients in this dataset is 63.4 (± 7.1) years. The dataset includes detailed lesion annotations, providing lesion coordinates and GG. Detailed lesion labelling and imaging variety make it a valuable dataset for model development.

Pi consists of a multi-stage system of deep learning, machine learning, and image processing algorithms that process mpMRI or MRI without intravenous contrast medium of the prostate, segmenting the prostate and identifying potential lesions calculating lesion and patient-level risk scores on a continuous 1–5 scale [3]. The AI outputs are intended for use by radiologists in either concurrent or second-reader reporting. PSA, age, or other clinical metadata are not included. More details on the model development are available in the Supplementary Material S2.

The validation data comprised the 42 latest cases that met the eligibility criteria at each of the five PAIR-1 sites, within the 30% of data held back from development, together with the 42 eligible cases from one site completely held out from development. The held-out site was chosen as it had the most ethnically diverse patient population and a scanner model not seen in AI model development to assess generalisation.

The decision to include 252 patients (42 per-site) for validation was based on a prospective sample size calculation to ensure a balanced representation across the sites.

### Statistical analysis

The pre-specified statistical analysis plan can be found in Supplementary Material S3.

The primary endpoint was the difference in diagnostic accuracy for detecting GG ≥ 2 at the patient level between MDT-supported radiologists and multiparametric Pi assessments, measured by receiver operating characteristic (ROC) area under the curve (AUC). A non-inferiority margin of 10% was set, with a one-sided significance level of 0.025 [13]. ROC AUCs were compared using Hanley and McNeil’s method [14] (95% confidence intervals obtained using bootstrapping).

Diagnostic accuracy at the patient level was evaluated using AUC, with sensitivity, specificity, and positive predictive value reported at Likert 3.5 for Pi and PI-RADS 3 for radiologists. If a PI-RADS score was missing, the radiologist’s Likert score (threshold of 3) was used instead, as Likert and PI-RADS scores perform equivalently for detecting GG ≥ 2 cancers [15].

The secondary endpoint was diagnostic accuracy for detecting GG ≥ 2 at the lesion level. We compared the original radiologist and AI assessments using free-response ROC (fROC) and ROC analysis. fROC analysis included radiologist-identified, biopsy-determined, and AI-identified lesions, plotting the number of FPs per patient. ROC analysis focused on lesions identified by the radiologist or biopsy, modelling AI as a confirmatory reader. A positive detection was defined by the overlap between AI-predicted and manually annotated lesions.

We also included three exploratory endpoints to compare the diagnostic accuracy of radiologists and AI across: (i) different field strengths, (ii) scanner ages, and (iii) hospitals. The analysis included patient and lesion performance per-site, using ROC curves. False negatives at the lesion level were identified to assess AI limitations.

## Results

### Clinical details

There were 1045 cases comprising 841 consecutive cases from the hospitals and 204 patients from the PROSTATEx dataset [10–12]. These were used either for model development (*n* = 793, 34% GG ≥ 2 cancers prevalence) or held back for validation (*n* = 252, 42 per-site, 31% GG ≥ 2 cancers prevalence) (Supplementary Fig. 1). The mean age of patients included for validation was 67.3 years (standard deviation: 8.5 years). The median pre-biopsy PSA was 6.81 ng/mL (interquartile ranges: 4.73–10.62 ng/mL).

A total of 137/252 (54.3%) patients from the validation set were biopsied: of them, 42/137 (29.9%) did not have any cancer, 17/137 (12.4%) had GG1, and 78/137 (57%) patients had at least one lesion GG2-5. A total of 96 GG ≥ 2 lesions were identified in the 78 patients, of which 58/96 (60%) were GG2, 16/96 (16.7%) GG3, 6/96 (6.25%) GG4 and 16/96 (16.7%) GG5 disease. The remaining 115/252 (45.6%) patients were not biopsied, and 90.4% of those had PSAD < 0.15 ng/mL^2^, meaning they were not at elevated risk of GG ≥ 2 [1]. All patients not biopsied were PI-RADS 1 or 2. Regarding the number of patients with PI-RADS 3, 9/27 (33.3%) patients had PSA > 0.15 ng/mL.

The validation dataset consisted of six scanners from two vendors (1.5 T and 3 T field strengths) (Supplementary Table 1).

The demographic characteristics of the PAIR-1 study participants are summarised in Supplementary Tables 2, 3 and 4.

### Diagnostic performance for detection of GG ≥ 2 cancers at patient level

**Fig. 2:**
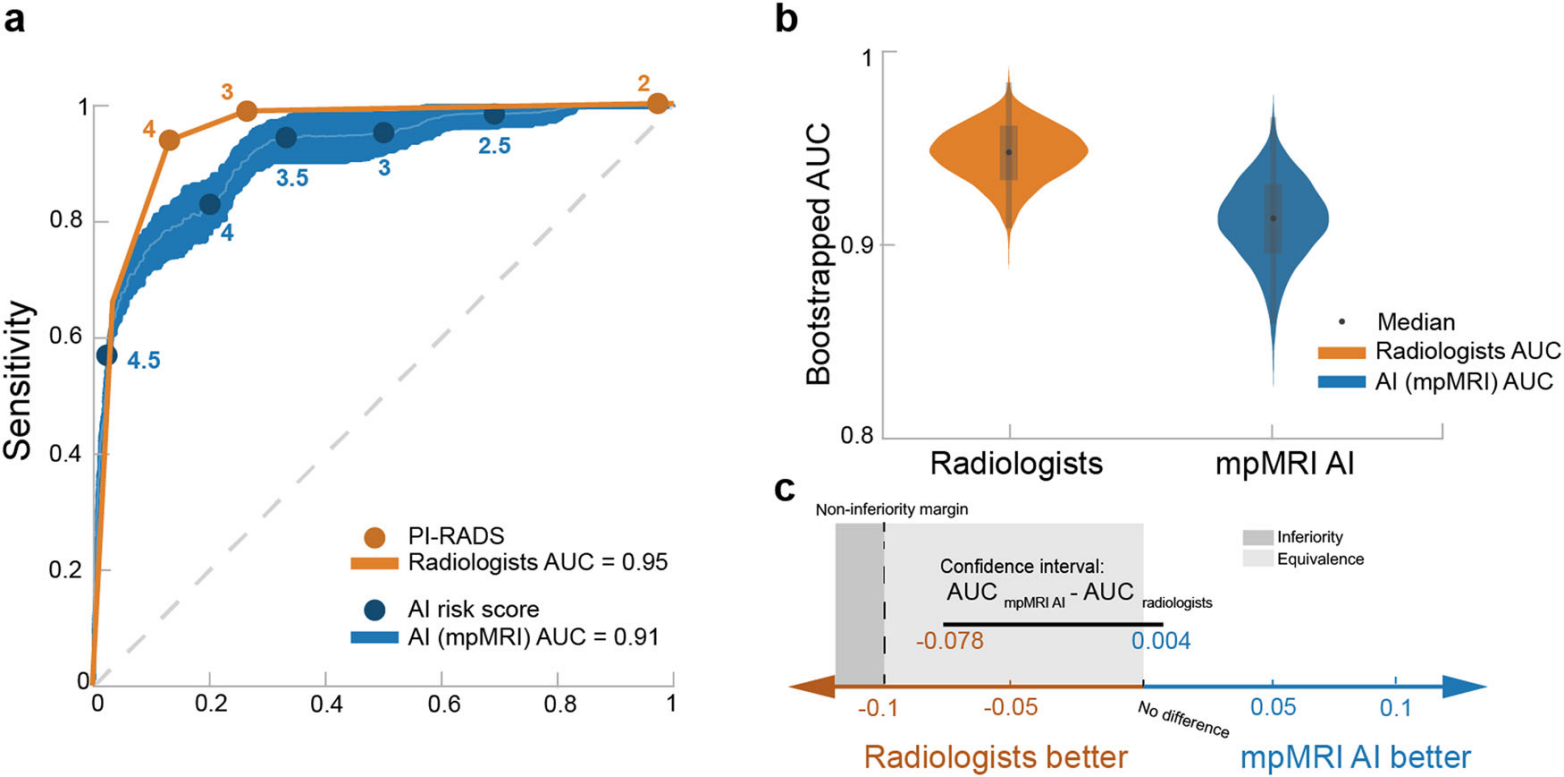
**a** ROC curves of the multiparametric AI model and radiologists at the patient level, displaying an AUC for the AI model of 0.91 (0.87–0.85), which is comparable to the radiologists’ AUC of 0.95 (0.92–0.97). Although radiologists achieved a higher AUC than the AI model, as illustrated by the bootstrapped AUC values in **b**, the difference in the ROC AUCs was statistically non-inferior based on the specified non-inferiority margin (*p* = 0.044) (**c**). In the violin plot, the box and whiskers show the data’s interquartile range and spread, while the width of the violin indicates the density of data points at different values (**c**). Visualisation of the mpMRI confidence intervals for (AUC _AI _− AUC _radiologists_), showing that the 97.5% one-sided confidence interval of –0.078 for (AUC_mpMRI AI_ – AUC_radiologist_) does not exceed the pre-specified non-inferiority margin of –0.1. Note that the two-sided 95% confidence intervals are shown here for visualisation, whereas the non-inferiority test was carried out in a one-sided manner

Figure 2a presents the ROC curve for the multiparametric AI model. The multiparametric AI model had AUC 0.91 (0.87–0.95) and radiologists AUC 0.95 (0.92–0.97). Non-inferiority is confirmed by the 97.5% one-sided confidence interval of –0.078 for (AUC_mpMRI AI_ – AUC_radiologist_), which does not cross the pre-specified non-inferiority margin of –0.1 (Fig. 2b, c).

The AI software’s primary threshold was established as 3.5. In the internal testing or holdout set from the development set, the AI model demonstrated a sensitivity of 96% (91–100%) and specificity of 68% (56–76%) at this threshold. With the validation set, the multiparametric AI exhibited a sensitivity of 95% (89–99%) and a specificity of 67% (60–73%). On the same data, the radiologists, at PI-RADS/Likert ≥ 3, identified csPCa with a sensitivity of 99% (96–100%) and specificity of 73% (67–80%). Table 1 shows the validation performance (sensitivity, specificity, and positive predictive values (PPV)) for different AI Likert thresholds.

**Table 1:** Validation (*N*_patients_ = 252) for the multiparametric AI model: Sensitivity, Specificity and PPV at different AI-Likert thresholds and expert radiologists at PI-RADS ≥ 3

	AI Likert ≥ 2.5	AI Likert ≥ 3	AI Likert ≥ 3.5	AI Likert ≥ 4	Radiologists PI-RADS ≥ 3
Sensitivity	0.99 (0.96–1.00)	0.95 (0.90–0.99)	0.95 (0.89–0.99)	0.85 (0.77–0.92)	0.99 (0.96–1.00)
Specificity	0.31 (0.24–0.38)	0.49 (0.41–0.56)	0.67 (0.60–0.74)	0.80 (0.74–0.86)	0.73 (0.67–0.80)
PPV	0.39 (0.33–0.46)	0.46 (0.38–0.53)	0.56 (0.48–0.65)	0.66 (0.56–0.75)	0.63 (0.54–0.71)

At the predetermined threshold of 3.5, PI’s sensitivity was 95% (89–99%) and specificity 67% (60–73%), a similar performance to expert radiologists at PI-RADS/Likert ≥ 3, which identified csPCa with a sensitivity of 99% (96–100%) and specificity 73% (67–80%)

Supplementary Fig. 2a shows the per-patient ROC analysis for identifying GG ≥ 2 cancers by site. The AI software performed well across all sites, with patient-level per-site AUC ≥ 0.83. Sites 1, 2, 4, and 5 showed high performance, with sites 3 and 6 having a slight drop in AUC. For the fully held-out site (Site 4), AI AUC was 0.92 (0.83–0.99). At the specified threshold of 3.5, the model’s performance metrics, including sensitivity and specificity, are consistent across most hospitals. However, two sites with lower AUC would benefit from a site-specific threshold to determine the significance of AI findings, address differences in equipment and imaging protocols, and provide a more optimum balance between sensitivity and specificity (Table 2).

**Table 2:** Overall sensitivity, specificity, and PPV for multiparametric AI model and expert radiologists at the patient level, as well as per-site performance for the AI model

Patient level		Sensitivity	Specificity	PPV	AUC
Overall	AI model	0.95 (0.90–0.99)	0.67 (0.60–0.74)	0.56 (0.48–0.65)	0.91 (0.87–0.95)
	Radiologists	0.99 (0.96–1.00)	0.73 (0.67–0.80)	0.63 (0.54–0.71)	0.95 (0.92–0.97)
Per-site (AI model)	Site 1	1.00 (1.00–1.00)	0.83 (0.65–0.96)	0.83 (0.67–0.96)	0.99 (0.95–1.00)
	Site 2	1.00 (1.00–1.00)	0.72 (0.55–0.87)	0.53 (0.29–0.77)	0.99 (0.96–1.00)
	Site 3	0.82 (0.57–1.00)	0.74 (0.59–0.90)	0.53 (0.28–0.77)	0.83 (0.67–0.97)
	Site 4	0.93 (0.77–1.00)	0.82 (0.68–0.96)	0.72 (0.50–0.92)	0.92 (0.83–0.99)
	Site 5	0.93 (0.75–1.00)	0.71 (0.54–0.88)	0.62 (0.40–0.83)	0.91 (0.78–0.99)
	Site 6	1.00 (1.00–1.00)	0.23 (0.09–0.37)	0.31 (0.17–0.47)	0.89 (0.74–0.98)

The AI model demonstrates similar performance metrics to radiologists when evaluated across several sites. At the specified threshold of 3.5, the model’s performance metrics, including sensitivity and specificity, are consistent across most hospitals. or the fully held-out site (site 4), AUC was 0.92 (0.83–0.99). However, sites 3 and 6 could benefit from a site-specific adjustment on the model threshold used, to enhance the model accuracy and reliability

Additional information regarding the exploratory endpoint comparing radiologists and AI diagnostic accuracy at a patient level at different field strengths and scanner ages can be found in Supplementary Material S4.

### Diagnostic performance for detection of GG ≥ 2 cancers at lesion level

83/96 lesions were identified in 71/78 patients using the multiparametric AI model at threshold 3.5. Radiologists identified 89/96 lesions in 77/78 patients at PI-RADS/Likert threshold 3. Therefore, the AI missed 14% of the lesions (13/96) across 9 patients, compared to 7% (7/96) missed by the radiologist across 6 patients.

**Fig. 3:**
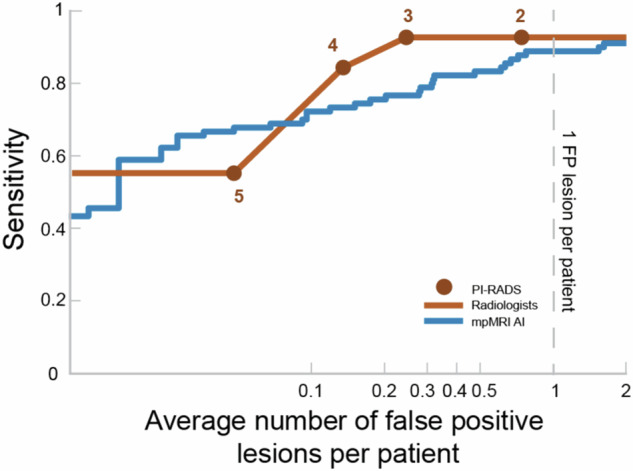
FROCs for multiparametric AI model and expert radiologists at lesion level, showing a higher average number of false-positive lesions for the AI model compared to radiologists at sensitivities above 70%. FP, false positive

Figure 3 shows the fROC analysis of the sensitivity of the original radiologists and AI at varying thresholds, compared to the average number of false-positive (FP) lesions. At one FP per patient, the original radiologists exhibited sensitivity 93% (88–98%) and mpMRI AI 89% (82–95%). The average number of FP lesions per patient for fROC analysis at specific thresholds for the AI model and PI-RADS is presented in Supplementary Table 5, showing a consistent lower number of FPs for the radiologist at both sensitivities of 80% and 90%.

Supplementary Figure 2b shows the per-lesion ROC analysis for identifying csPCa, at lesion locations identified by the original radiologist, stratified by site. There was a broader range of AUC values in comparison to the AUC values at the patient level, with two sites showing a noticeably lower AUC and an additional site with a slight drop. Despite this, the AUC for the unseen site remained high at the lesion level, with a value of 0.92.

At the specified threshold of 3.5, the AI model demonstrates an overall performance comparable to that of radiologists. However, per-site analysis reveals high sensitivity and specificity for three sites, while the other three show lower performance. These variations underscore the need for site-specific adjustments on the model threshold to improve the models’ accuracy (Supplementary Table 6).

In-depth details on the AI false negative lesions can be found in Supplementary Material S5.

## Discussion

We compared a CE-marked DL-CAD medical device with the performance of the original MDT-supported radiologists, using prostate MRI data from different hospitals. Both were compared against annotations of biopsy results mapped onto the MRI scans by expert radiologists independent of the original reporting radiologists. At a per-patient level on the separated internal validation dataset, Pi was non-inferior to the MDT-supported radiologists based on the pre-specified non-inferiority margin. At the predetermined suspicion threshold of 3.5, Pi’s sensitivity was 95% and specificity 67%, while the original radiologists at PI-RADS/Likert ≥ 3 had a sensitivity of 99% and specificity of 73%.

If high specificity can be replicated in general use while maintaining sensitivity, DL-CAD may enable reductions in biopsies and associated costs without missing a significant additional number of men with GG ≥ 2 cancers. An international panel of radiologists has identified this software requirement as essential [16].

Academic and commercially available AI software have shown comparable performance in distinguishing between radiologists and DL algorithms [8], with most studies being done on single-centre datasets, which hinder knowledge of the generalisability of predictive models [17]. Our study adds to emerging multi-centre, multi-vendor, and multiple MRI-field strength validation of AI for PCa detection [18, 19]. The PI-CAI investigators evaluated a confederated AI of the 5 top-performing DL algorithms participating in a challenge [18]. The training, tuning, and testing for GG ≥ 2 cancers were from four Northern European centres. When evaluated against the original radiologists who were involved in biopsy decisions, the AI performance was found to be non-inferior. The study of Cai et al also noted that the performance of their DL model developed on multi-vendor data was not different from radiologists for the detection of csPCa [19]. In our study, using a diverse dataset with both 1.5 T and 3 T scanners, Pi was non-inferior to radiologists working in MDTs (*p* = 0.044).

The good patient-level performance of Pi per-site indicates promising generalisation, with AUC ≥ 0.83, compared to the AUC of 0.91 of the pooled data from all sites. In the fully held-out site, Pi had an AUC of 0.92. The lower AUC at two sites indicates a need for site-specific adjustments on the AI model threshold to improve the models’ accuracy as also noted by Netzer et al [17].

Studies such as PROMIS, MRI-FIRST, and 4 M have provided valuable insights into the role of MRI in diagnosing PCa, with reported sensitivities of 88%, 94%, and 93%, and specificities of 45%, 30%, and 68%, respectively [20–22]. Specificity in this context refers to the ability of the diagnostic test to correctly identify patients who do not have the disease, thereby reducing FP results. Higher specificity is particularly important in clinical practice as it can lead to a significant reduction in unnecessary biopsies. For instance, a specificity of 68%, as observed in the 4 M study, suggests a more reliable exclusion of non-cancer cases, which could directly translate to fewer invasive procedures, reduced patient anxiety, and lower healthcare costs. This increased specificity highlights the potential of MRI as a valuable triage tool, allowing clinicians to better target those who would truly benefit from a biopsy while sparing others from the procedure.

While the specificity of Pi is high at the patient level, at the lesion level, it exhibited a higher number of FPs (i.e., higher risk of recommending biopsy for non-cancer or GG1 lesions). Our number of FPs for lesions ≥ 3 was similar to Hosseinzadeh et al [23] but within an acceptable range for software in this class [24]. Pi also missed 13/96 (14%) GG ≥ 2 lesions in 9 patients compared to radiologists who missed 7/96 (7%) lesions. Clinical implementation must, therefore, consider the potential for AI to over-detect or miss lesions. Pi is not intended as a stand-alone lesion-level biopsy targeting application but is a decision-support tool to assist radiologists based on their experience as well as on clinical assessments in an MDT environment. Prospective studies are required to determine the optimal clinical approach to additional AI-identified lesions, balancing the harm and costs associated with additional targets (potential additional detection of both clinically indolent and csPCa) based on urological preferences [25].

Pi is a fully automated software that fits into existing workflows, without requiring radiologists to change systems or manually copy data. It runs automatically and integrates with the Radiology Information System patient lists, enabling users to prioritise reporting.

Segmentation identifies key anatomical regions using a deep learning-based AI algorithm. The software applies machine learning AI models to determine regions of interest for cancer as well as the patient’s overall risk of PCa. The software then prepares a template report and other formats of results for subsequent analysis and integration by the radiologist.

Deployment of the technology can be done remotely, and radiologist training can similarly be done via an online platform, and all instructions for use and the training manual are provided in digital format.

This study has limitations. First, whole-mount pathology (i.e., this study relies on needle biopsy data) was not used in model development, but the prostatectomy standard results in selection bias as only surgically fit men with intermediate-risk disease undergo surgery. Secondly, 46% of patients did not receive a biopsy, and only radiologist-identified lesions were targeted. The patient-level and lesion-level ground truth likely misses some cancers, impacting the sensitivity and specificity of both radiologists and AI. Using the EAU 2024 guidelines [26] PSAD threshold of < 0.20 ng mL for biopsy of MRI negative cases, 95.7% of the patients would no longer be considered at elevated risk for GG ≥ 2 disease [27]. This approach to model development and testing is similar to that adopted by Cai et al in their multi-vendor study [19], where negative MRI scans also did not undergo biopsy afterwards for verification, and the PSAD of their negative cases was unknown. In the PI-CAI study, 59% of patients with negative MRI scans did not undergo biopsy, and although their PSAD is unknown, a 2-year follow-up for negative cases was followed [18]. Patients with a negative MRI avoiding biopsy should adhere to their healthcare practitioners’ recommended guidelines for future PSA screening, based on individual risk level [28]. Thus, the AI/radiologists comparison rather than absolute performance was the focus of these multi-centre, multi-vendor analyses. We plan to test non-inferiority in a prospective study with AI lesions biopsied independently of radiologist-identified regions.

The choice of a 10% non-inferiority margin could also be seen as a potential limitation and larger cohorts to support smaller margins (e.g., the PI-CAI study [18], had a 5% margin) should be the next step for more robust validation. Another limitation of our study is that the development and separate validation datasets were from the same population groups, and performance may change for populations with different ethnicities, disease prevalence, and age groups. It is also important to highlight that the scanners included in our study do not accurately reflect the entire market share of MRI scanners.

It is crucial to emphasise the importance of optimal image quality for proficiency in AI, with most models and AI evaluations excluding patients with poor quality [29–32]. Furthermore, we excluded patients with a previous history of PCa, and the performance of Pi for active surveillance is unknown. Lastly, our study used the public dataset PROSTATEx for AI development. While the dataset’s size is advantageous for developing an AI, it does not follow PI-RADS MRI acquisition guidelines [33], which might affect the model’s generalisation to datasets adhering to modern PI-RADS standards.

## Conclusion

This study evaluates an AI medical device for prostate MRI across multiple centres and vendors, showing accuracy comparable to MDT-supported radiologists. AI software could enhance the MRI pathway for biopsy decisions. Prospective diagnostic accuracy studies and randomised controlled trials with AI-identified lesions biopsied independently will determine the optimal synergy between AI software and medical experts for personalised PCa diagnosis in MDT settings.

## Supplementary information

ELECTRONIC SUPPLEMENTARY MATERIAL

## Abbreviations

AUC: Area under the curve
AI: Artificial intelligence
CE: Conformité Européenne
DL: Deep learning
DL-CAD: Deep-learning-based computer-aided detection
GG: Gleason Grade Group
MRI: Magnetic resonance imaging
MDT: Multidisciplinary team
PCa: Prostate cancer
PI-RADS: Prostate Imaging-Reporting and Data Systems
PSA: Prostate-specific antigen
PSAD: Prostate-specific antigen density
