# Supplementary material for: AI-powered prostate cancer detection: a multi-centre, multi-scanner validation study
Source: Eur Radiol. 2025 Feb 28;35(8):4915–24. doi: 10.1007/s00330-024-11323-0 (PMC12226644; doi:10.1007/s00330-024-11323-0)
Supplement: Supplementary file 1 — ELECTRONIC SUPPLEMENTARY MATERIAL [file 330_2024_11323_MOESM1_ESM.docx]

**Supplementary Material S1: Reading strategy**

The methodological approach to identify lesions from the MRI and biopsy reports and mapping to AI readings, is described below:

1. **Data collection:**
   - **MRI reports:** Reports were collected from several NHS hospitals, containing detailed assessment by clinical radiologists and PI-RADS/Likert scores.
   - **Biopsy reports:** When available, biopsy reports were collected, providing histopathological confirmation and grading of lesions.
2. **Expert annotations:**

- A team of expert radiologists manually marked lesions identified in the MRI reports on MRI images and cross-referenced them with the biopsy reports.
- For each identified lesion, PI-RADS score from the MRI report and the Gleason scores were annotated. For lesions only reported in the biopsy reports, the expert radiologists assigned PI-RADS 0.
- While annotating cases, expert radiologists conducted an image quality check and excluded cases based on previously defined exclusion criteria. The reason for exclusion was annotated and no scores of image quality were recorded.

1. **Quality control of annotations:**

- All annotations on the separate validation set were independently verified by another expert genitourinary radiologist to ensure that the spatial location of these lesions was accurate, as well as their histopathological data when available.

1. **Data Analysis:**

- **Patient-level:** The risk score is determined by the maximum score across all voxels within the prostate area, similar to a classification task. For positive cases, this score matches the maximum risk score across all predicted lesions, regardless of whether this score is at the index lesion or another lesion. If a patient has a risk score above the AI model's threshold, then that patient is considered to have csPCa by the AI.
- **Lesin-level:** The risk score is derived from the maximum score within the predicted lesion’s segmentation area.
- **Matching with ground truth:** To map AI-predicted lesions with ground truth lesions annotated by expert radiologists, an AI-predicted lesion is considered a match with a ground truth lesion if they overlap.

**Supplementary Material S2: Model development**

A total of 793 cases (with a 34% prevalence of csPCa) were used for model development, consisting of 589 cases from five NHS hospitals and 204 cases from the public dataset PROSTATEx. The data was partitioned into three sets: training, tuning, and internal testing, each with an 34%, 37% and 33% prevalence of csPCa, respectively.

The Pi model consists of a multi-stage system of deep learning, machine learning and image processing algorithms that process a multiparametric MRI (mpMRI) or MRI without intravenous medium contrast of the prostate. Imaging data were preprocessed before feature extraction. Images were resampled to the same grid, registered to the T2w axial image space and normalised. Subsequently, the model segmented the prostate, identified potential lesions, and calculated lesion and patient-level risk scores on a continuous 1-5 scale. Following training, the model’s threshold was selected based on the combination of the training and tuning sets to achieve an operating point with 0.95 sensitivity. The model was then evaluated on the internal testing set and in a separate validation set with 252 cases from six NHS sites from PAIR-1.

Model performance during training and evaluation was assessed using sensitivity, specificity, AUC, negative predictive value and positive predictive value at predetermined threshold. Confidence intervals were obtained through bootstrapping with 2000 replications/iterations.

**Supplementary Material S3: PAIR-1 Statistical Analysis Plan**

**
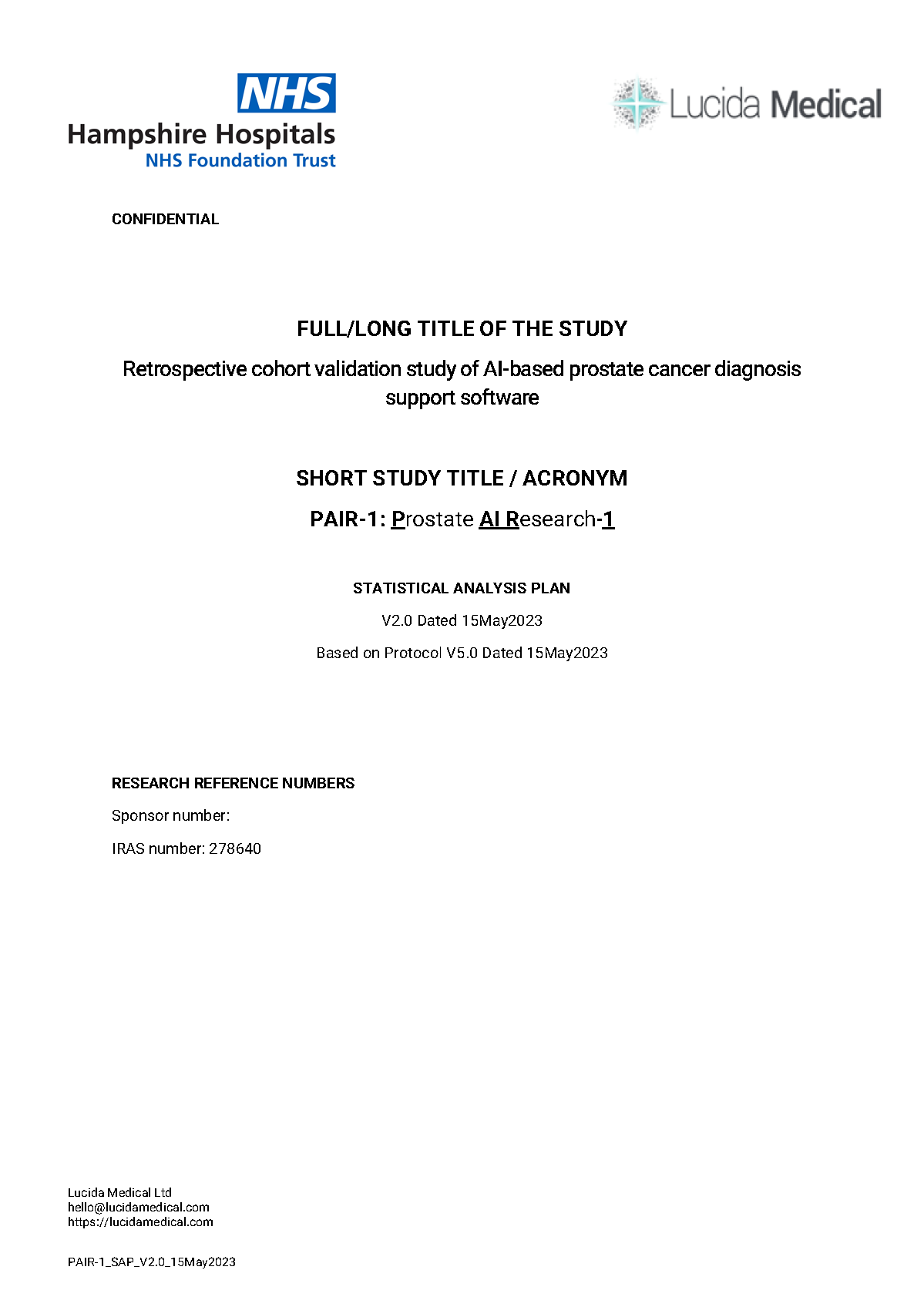
**

**
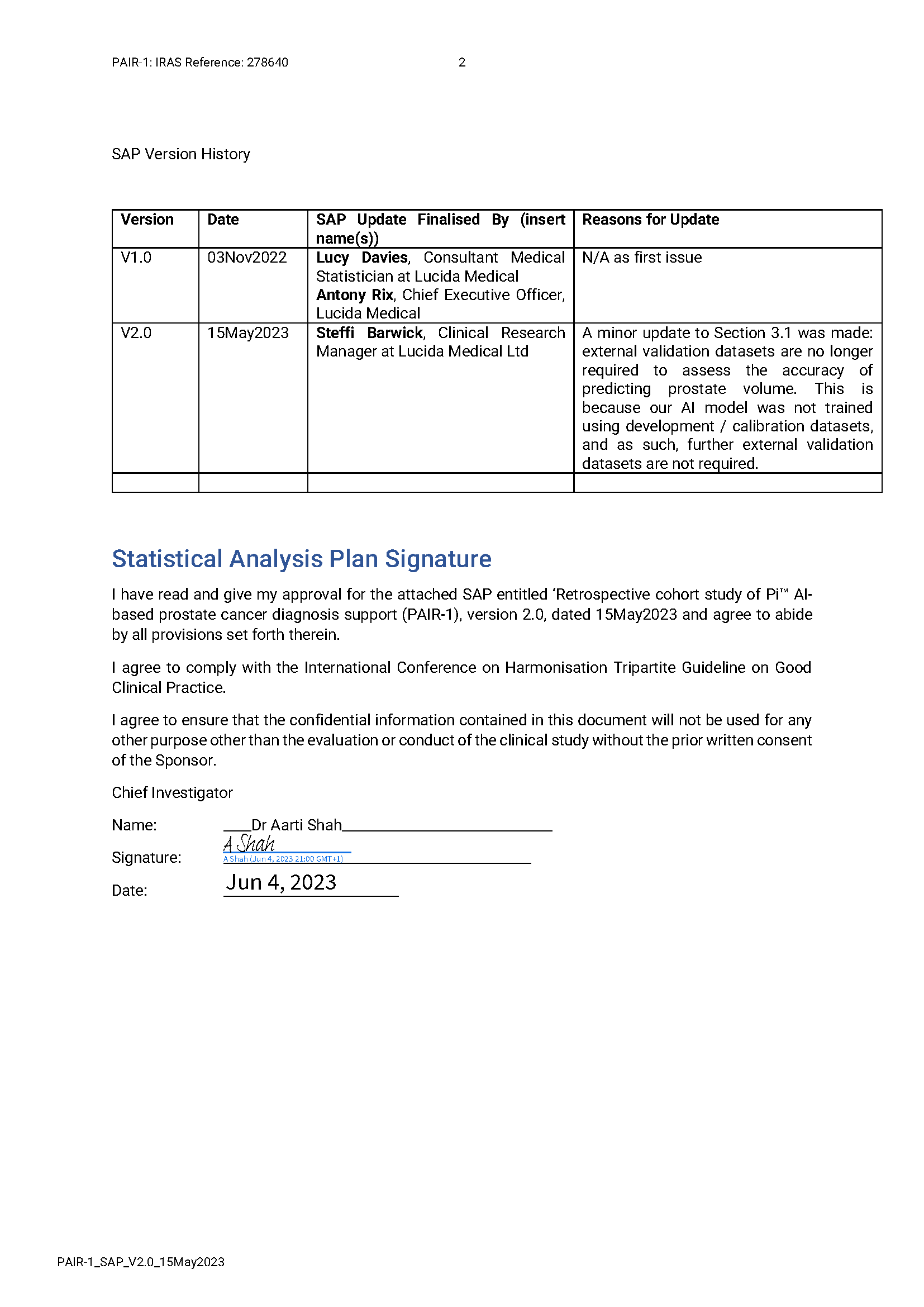

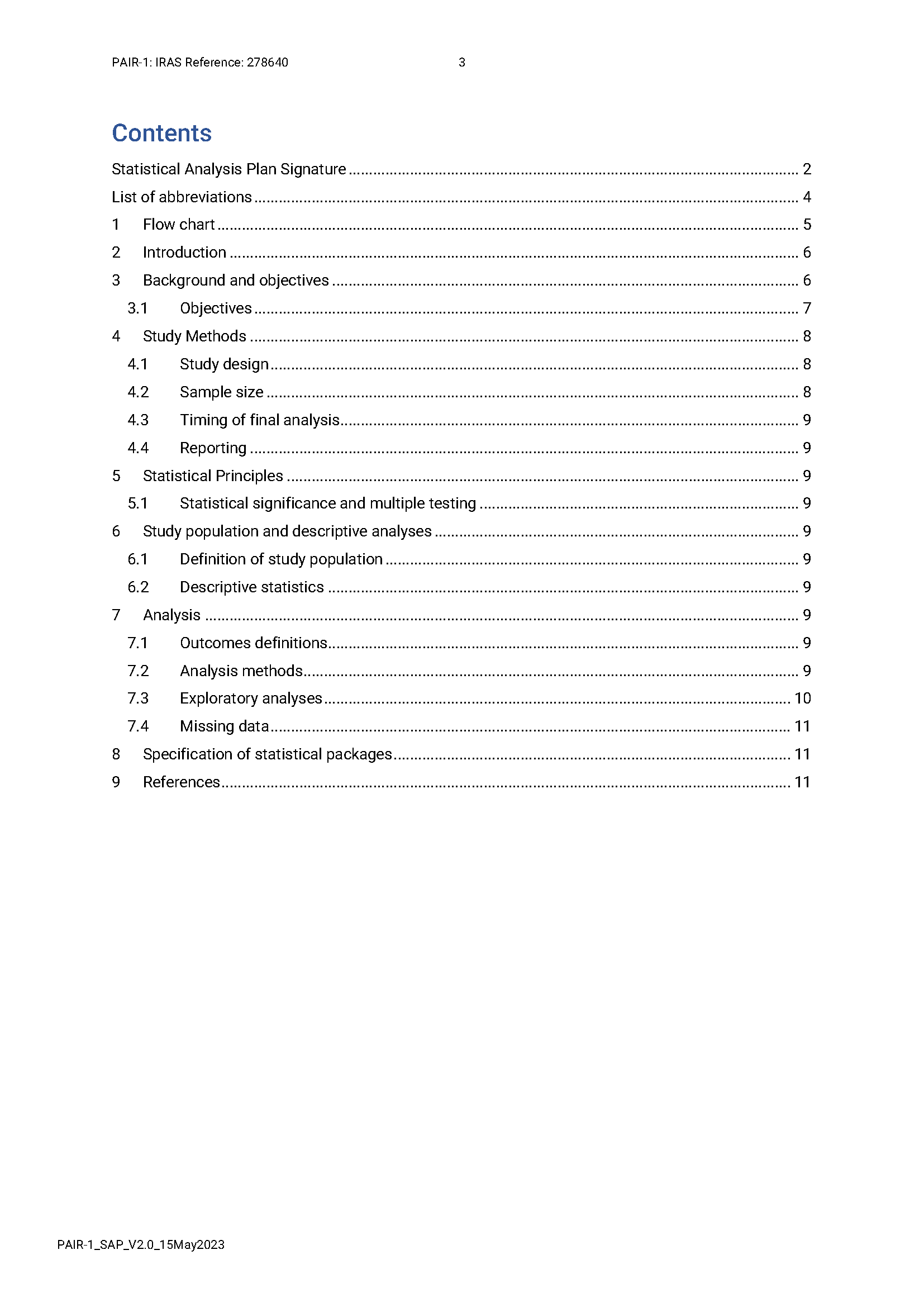
**

**
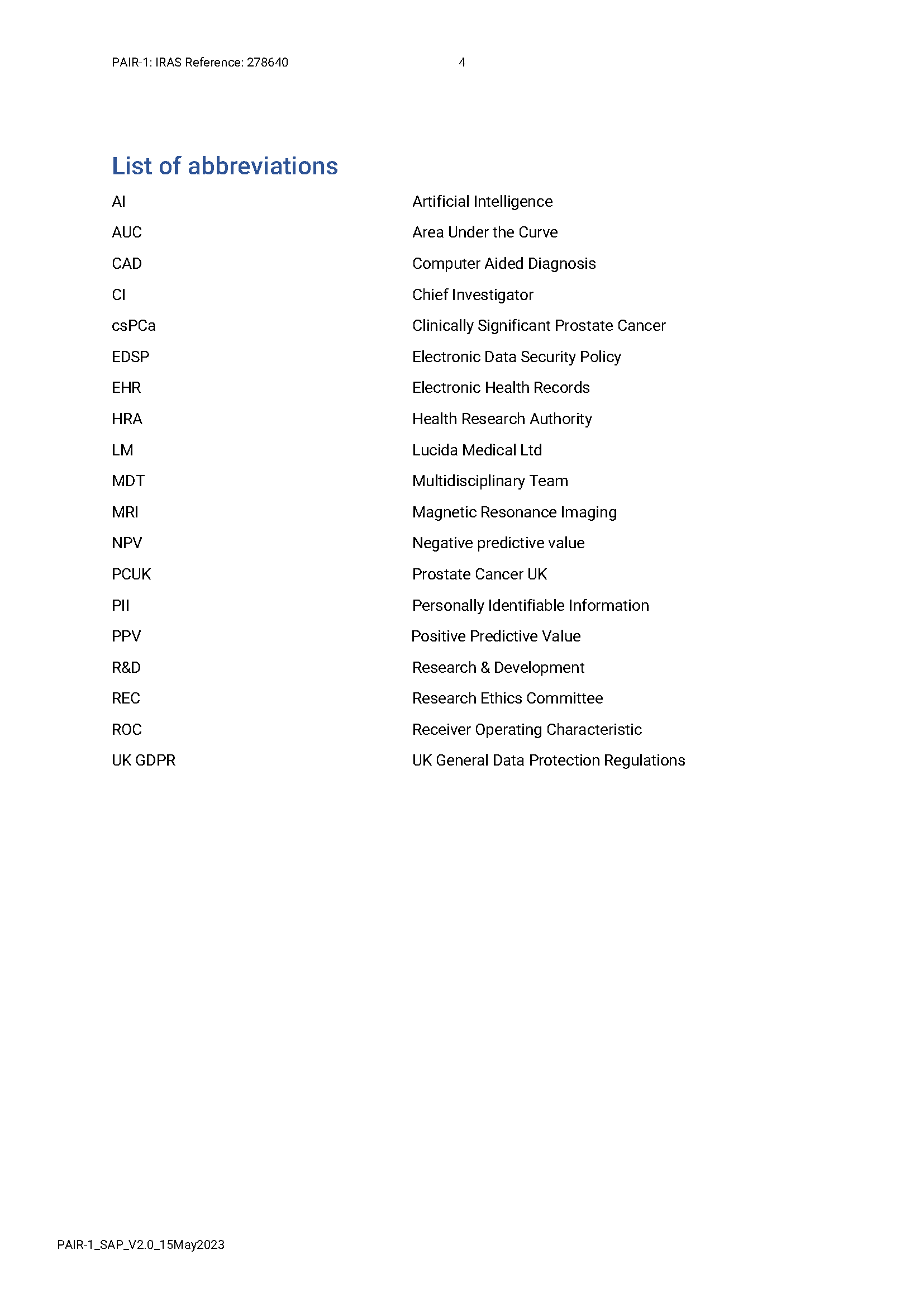
**

**
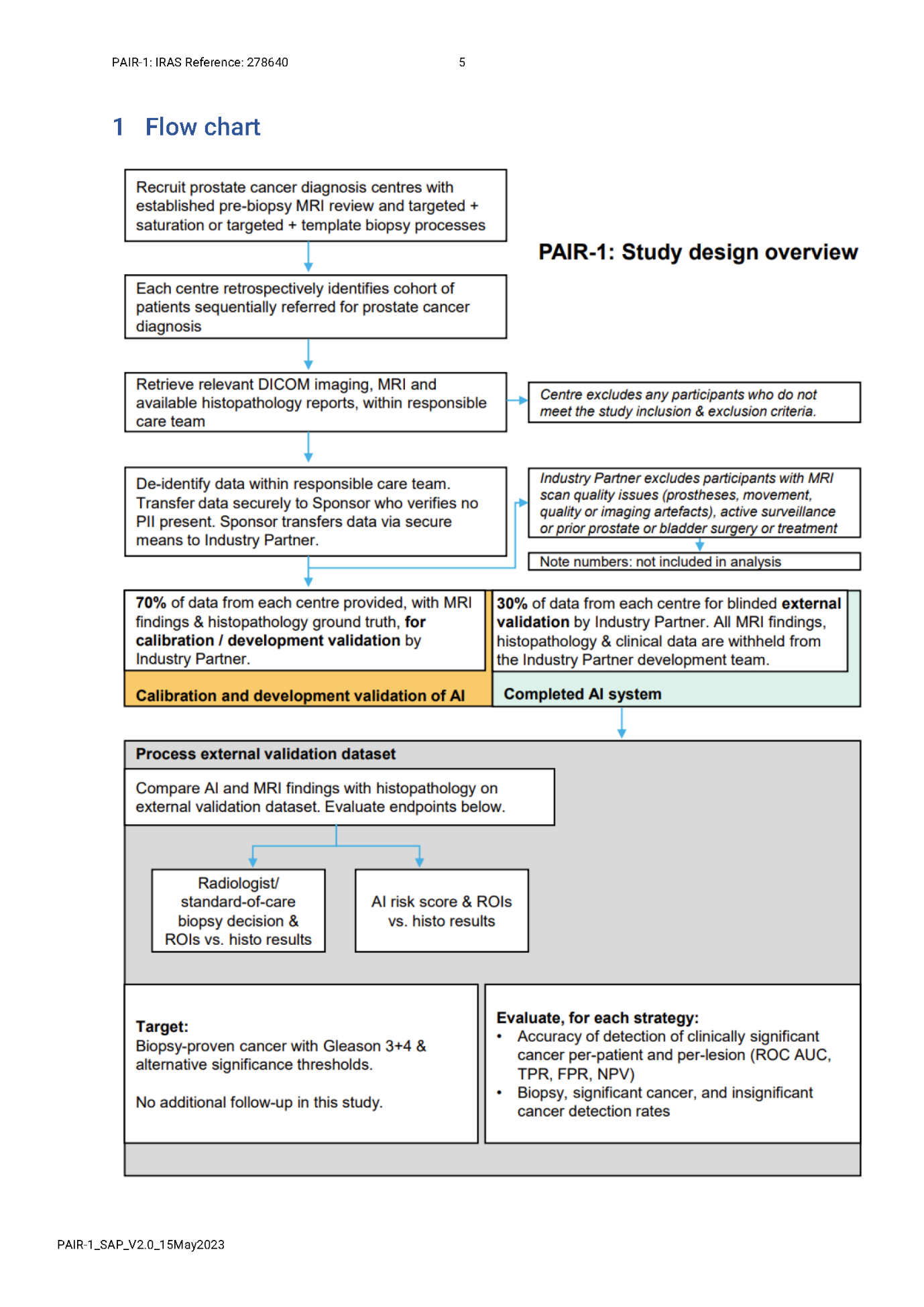
**

**
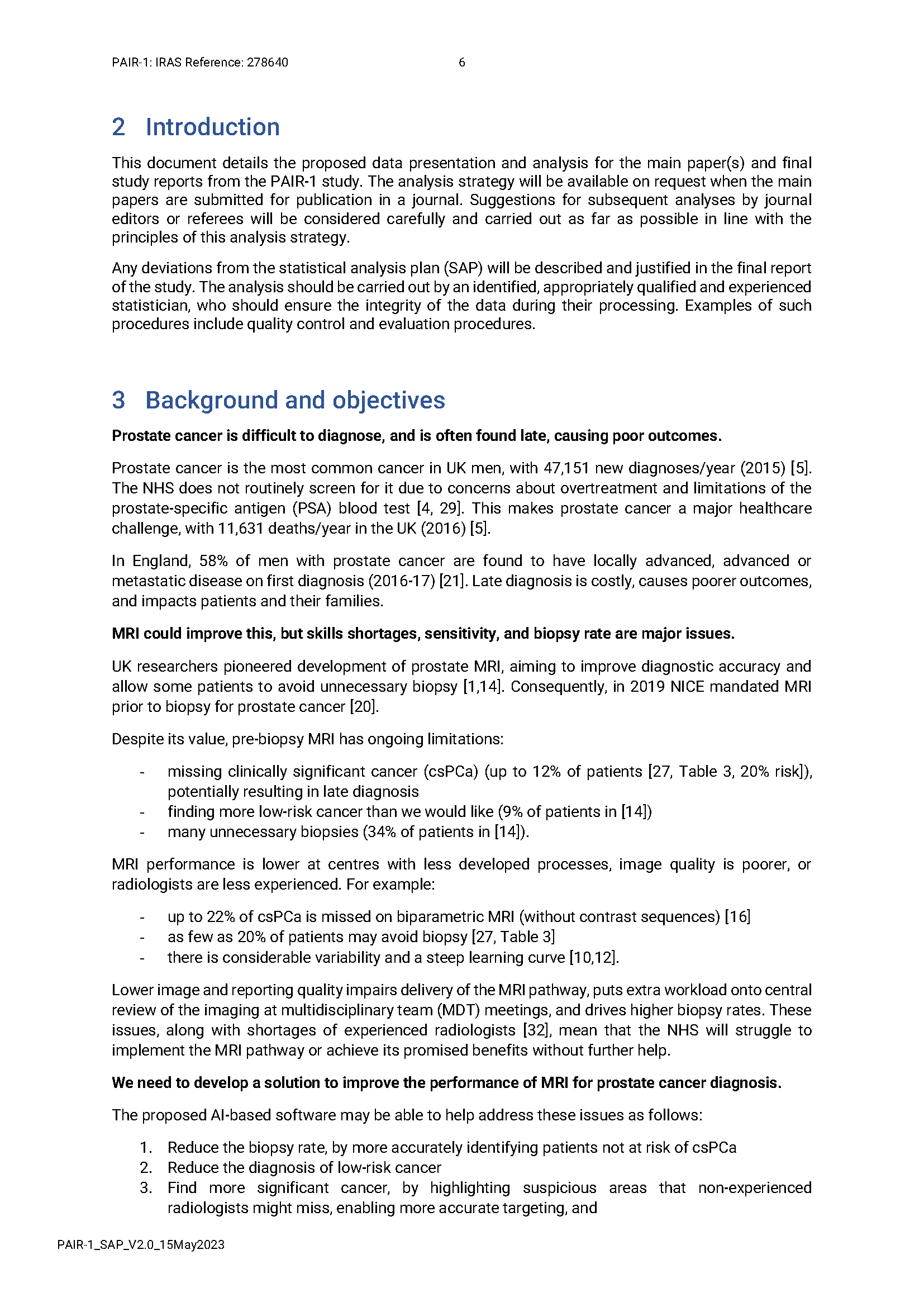
**

**
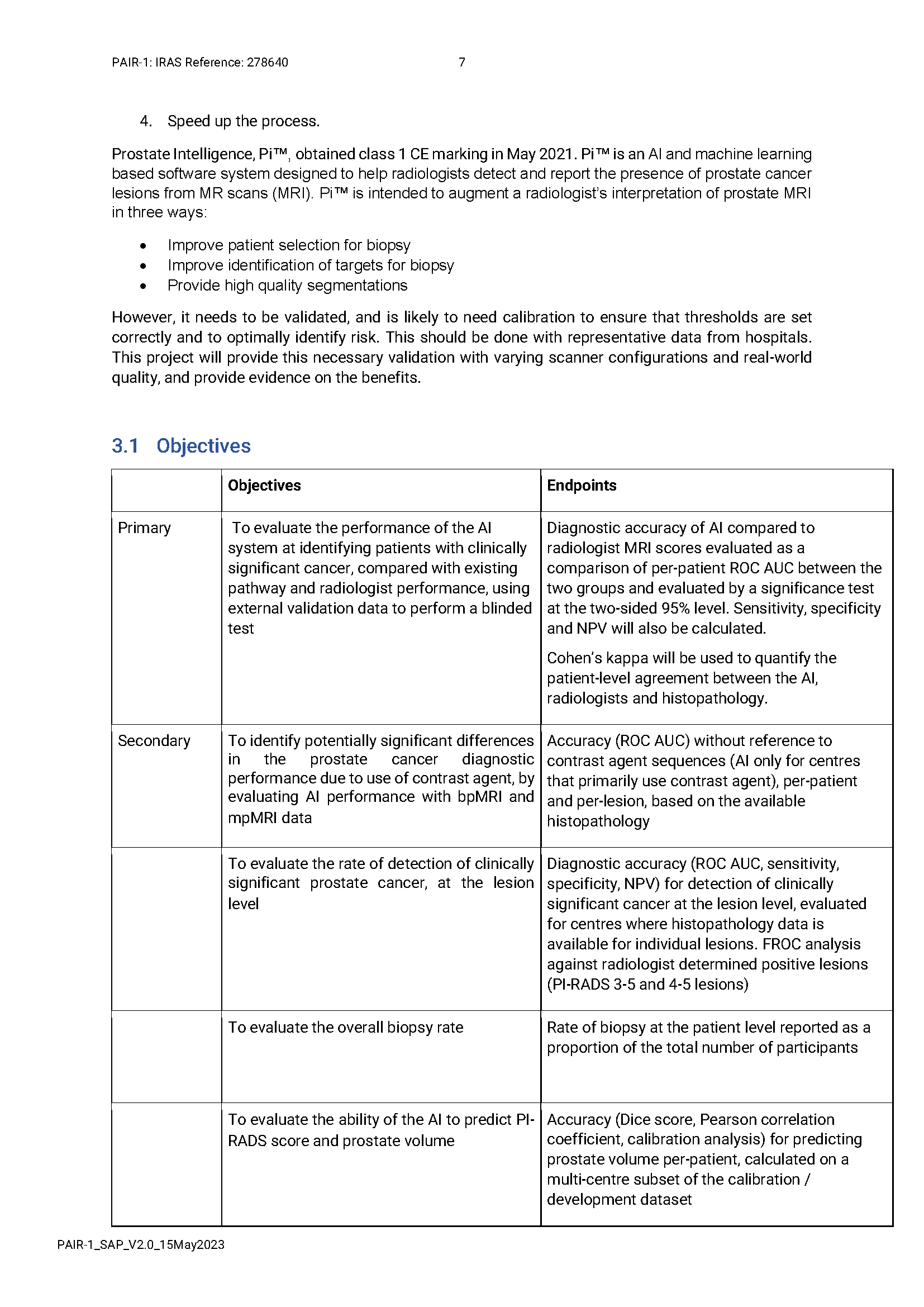
**

**
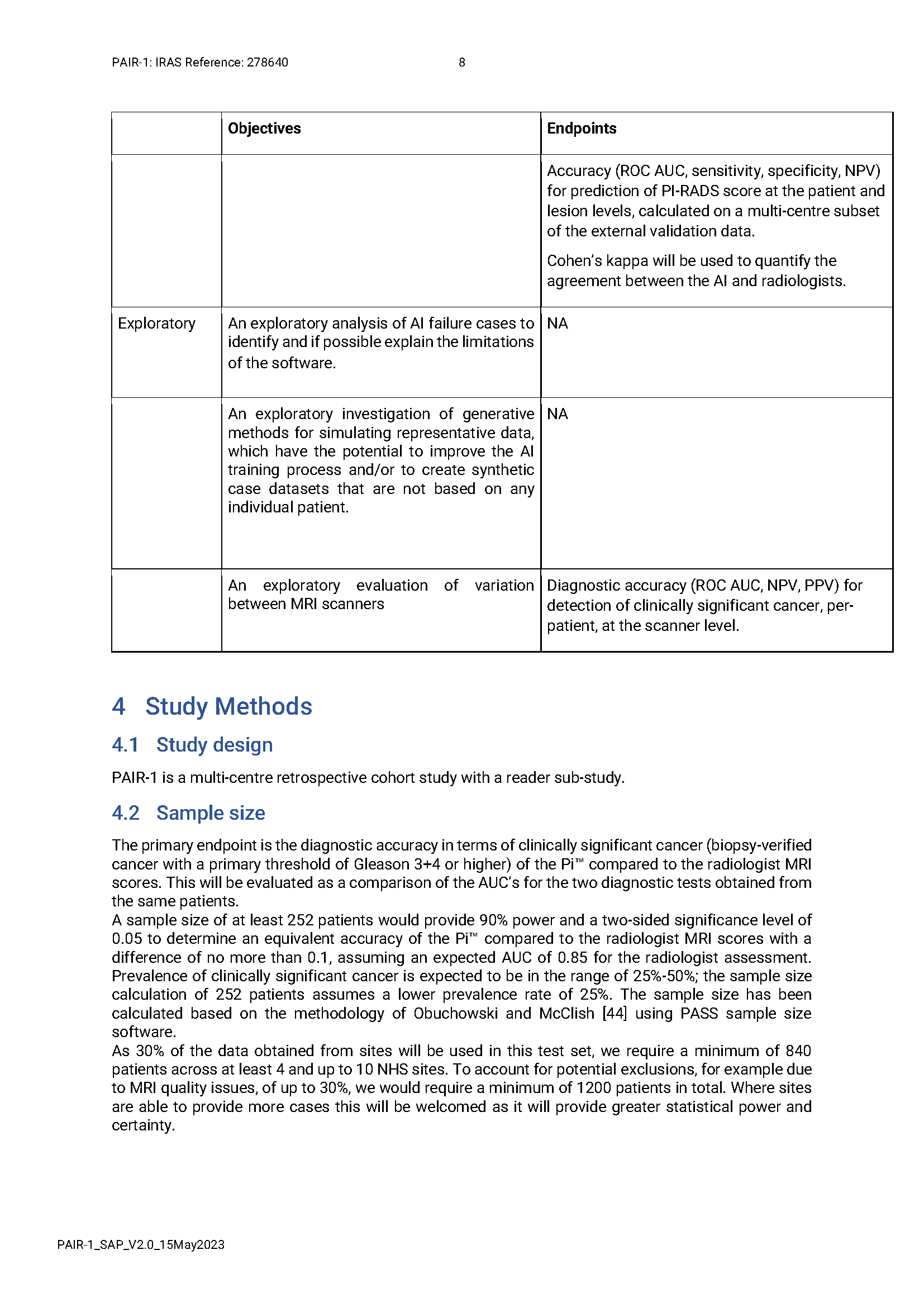
**

**
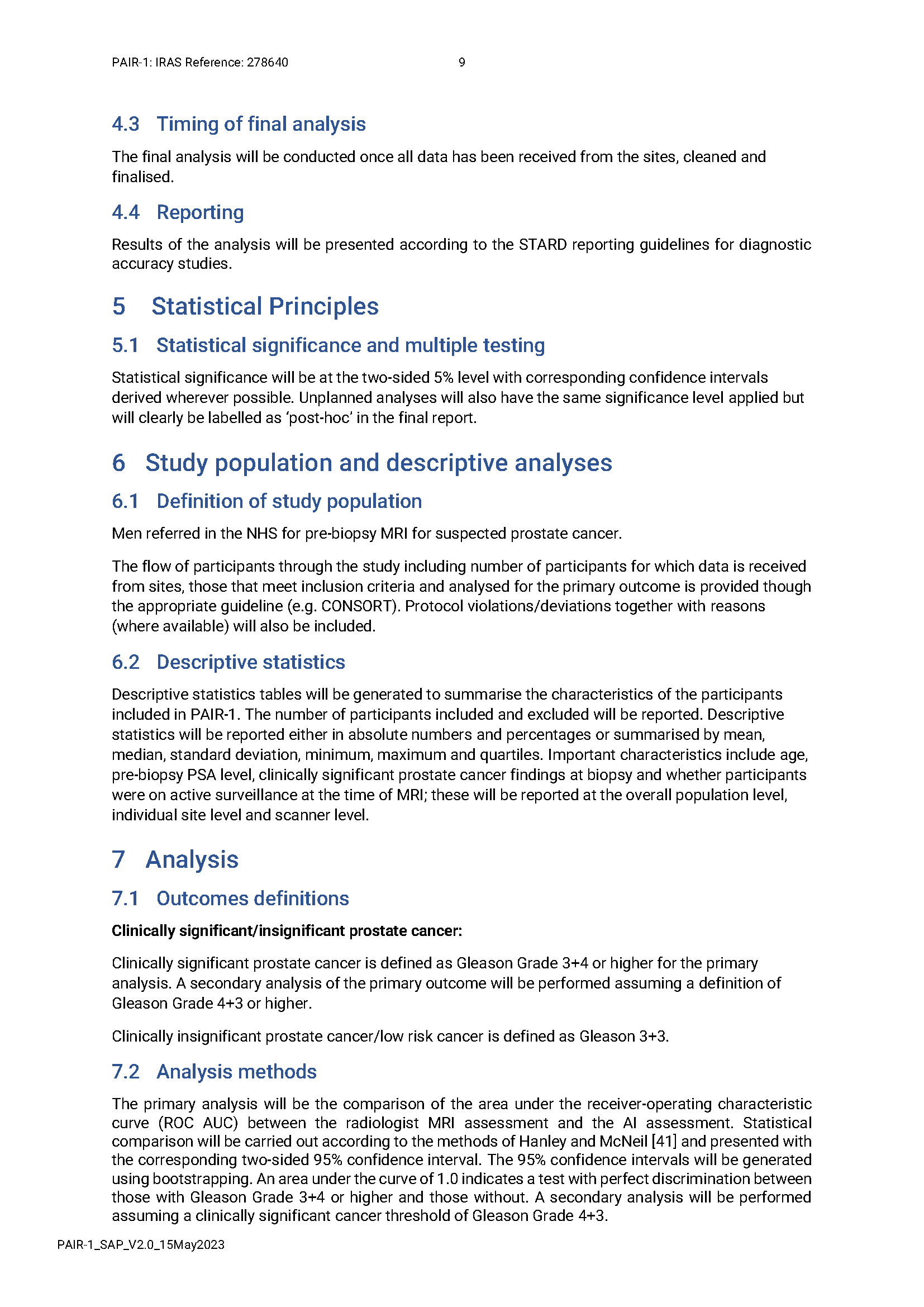
**

**
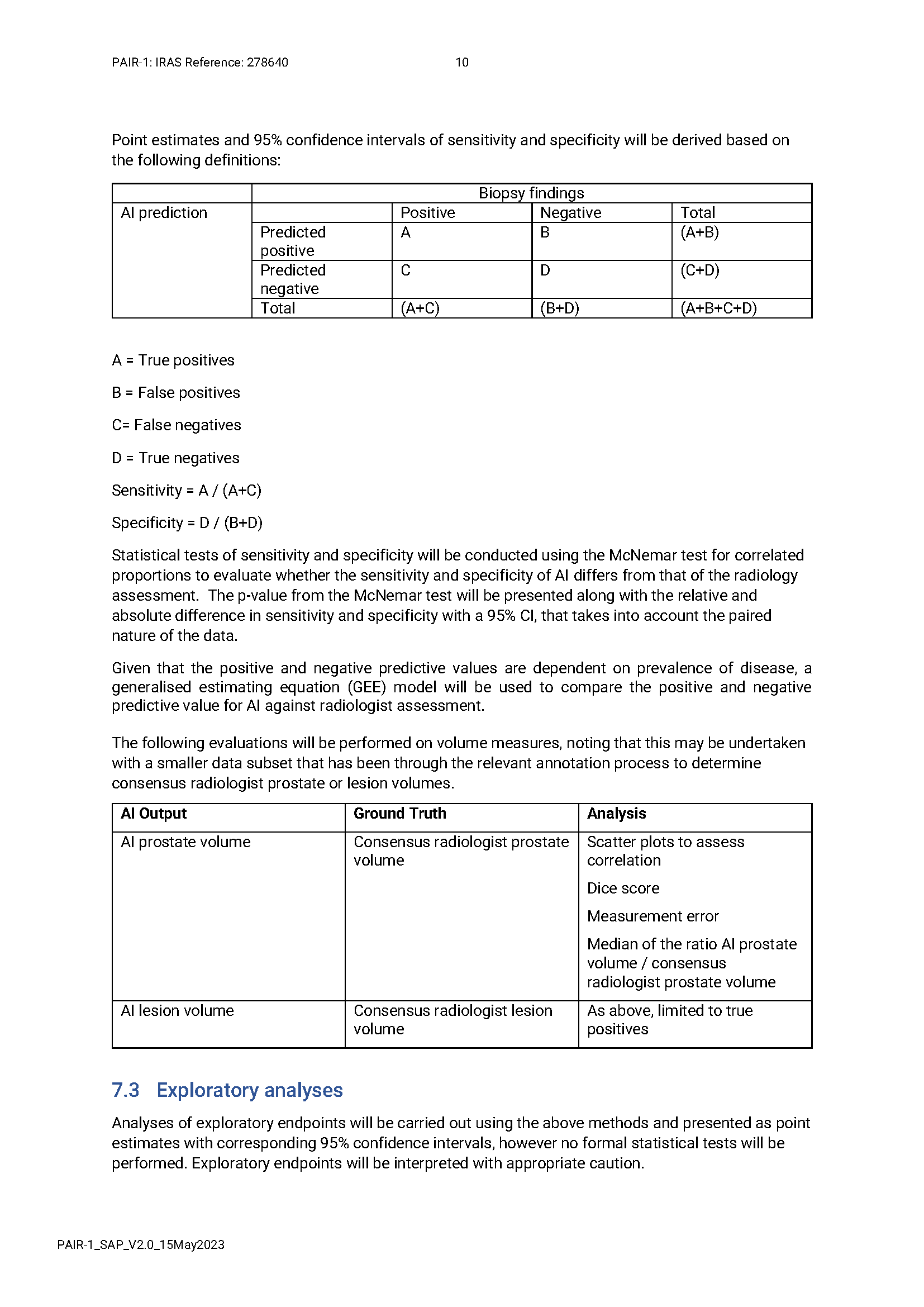
**

**
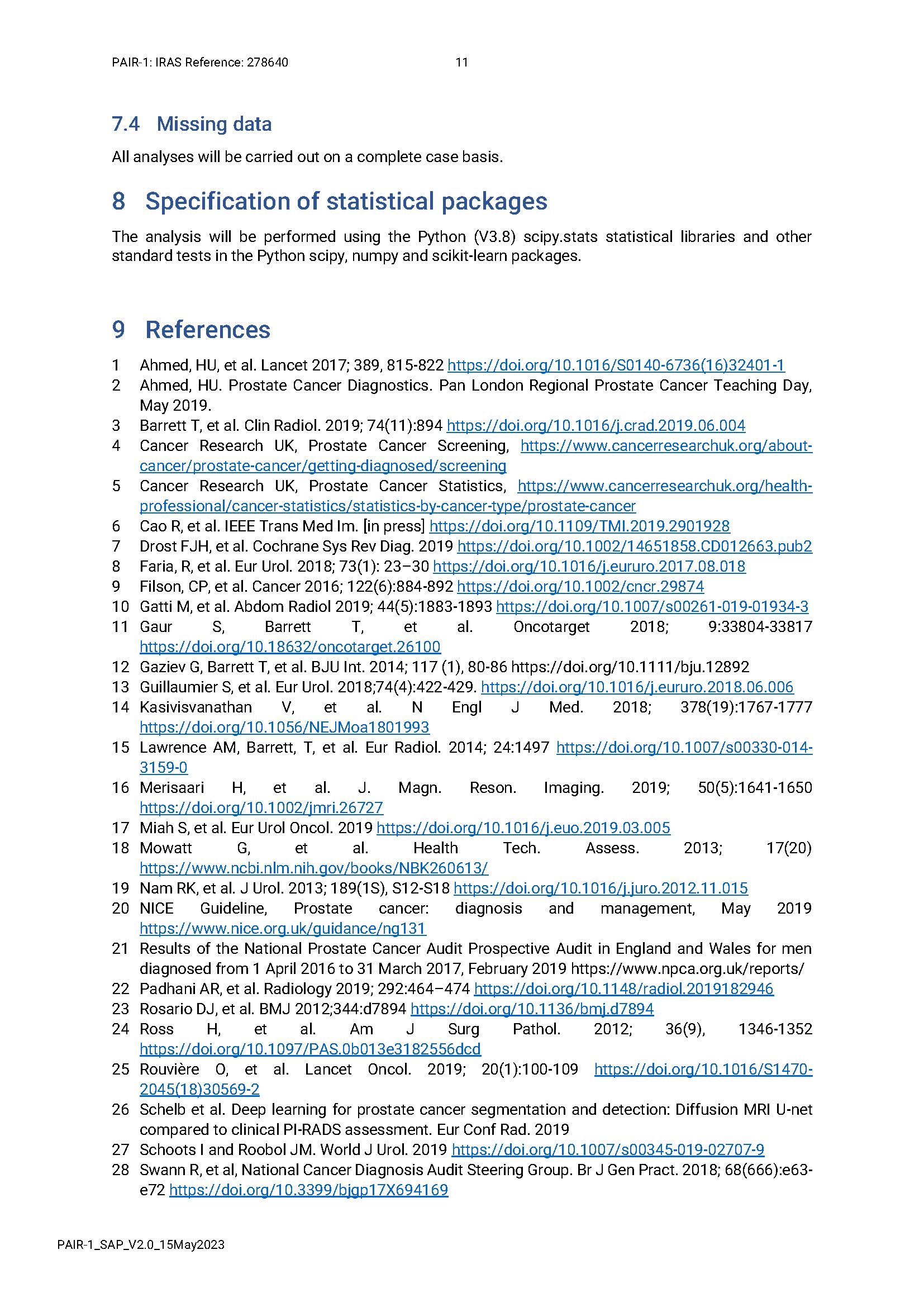
**

**
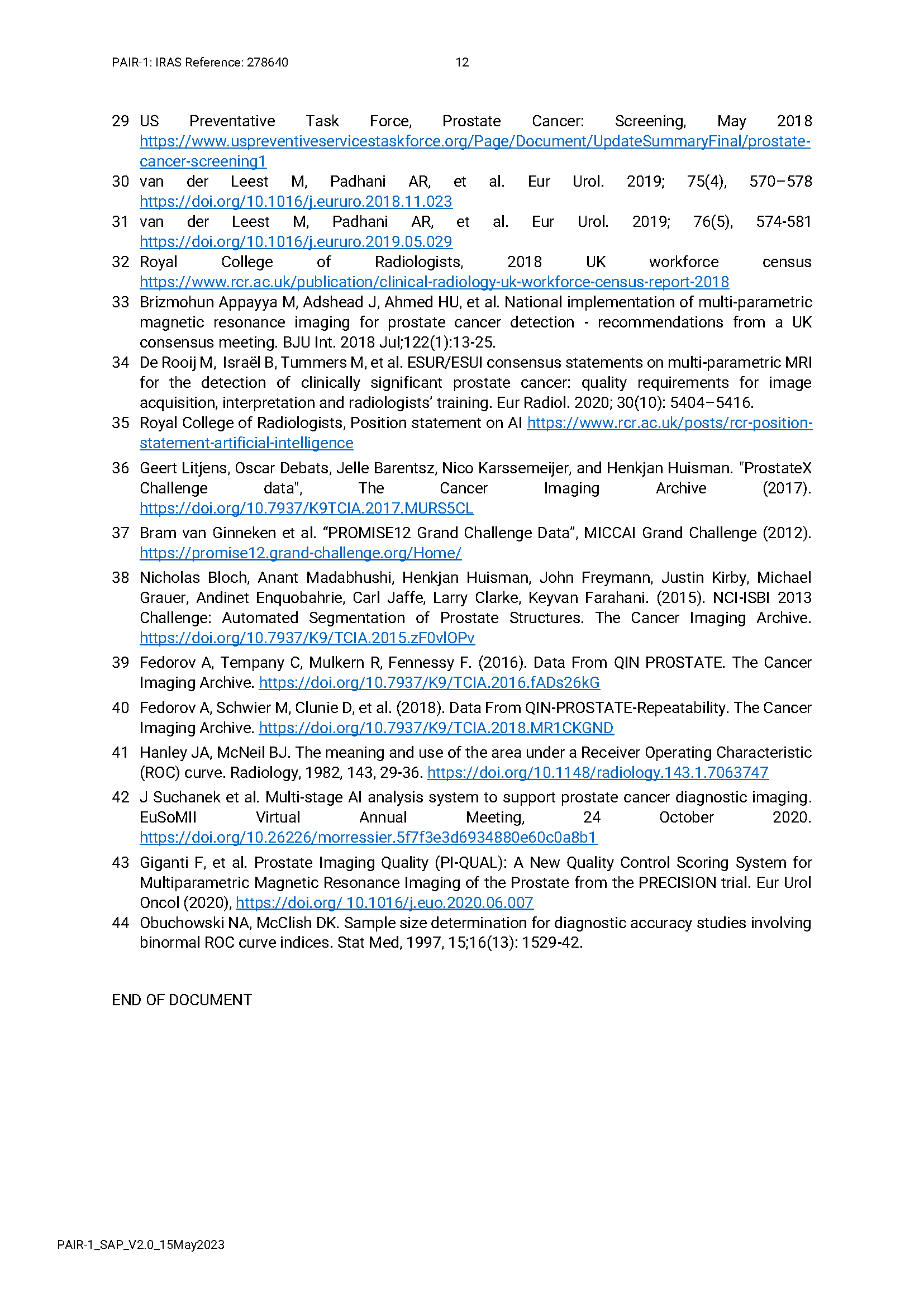
**

**
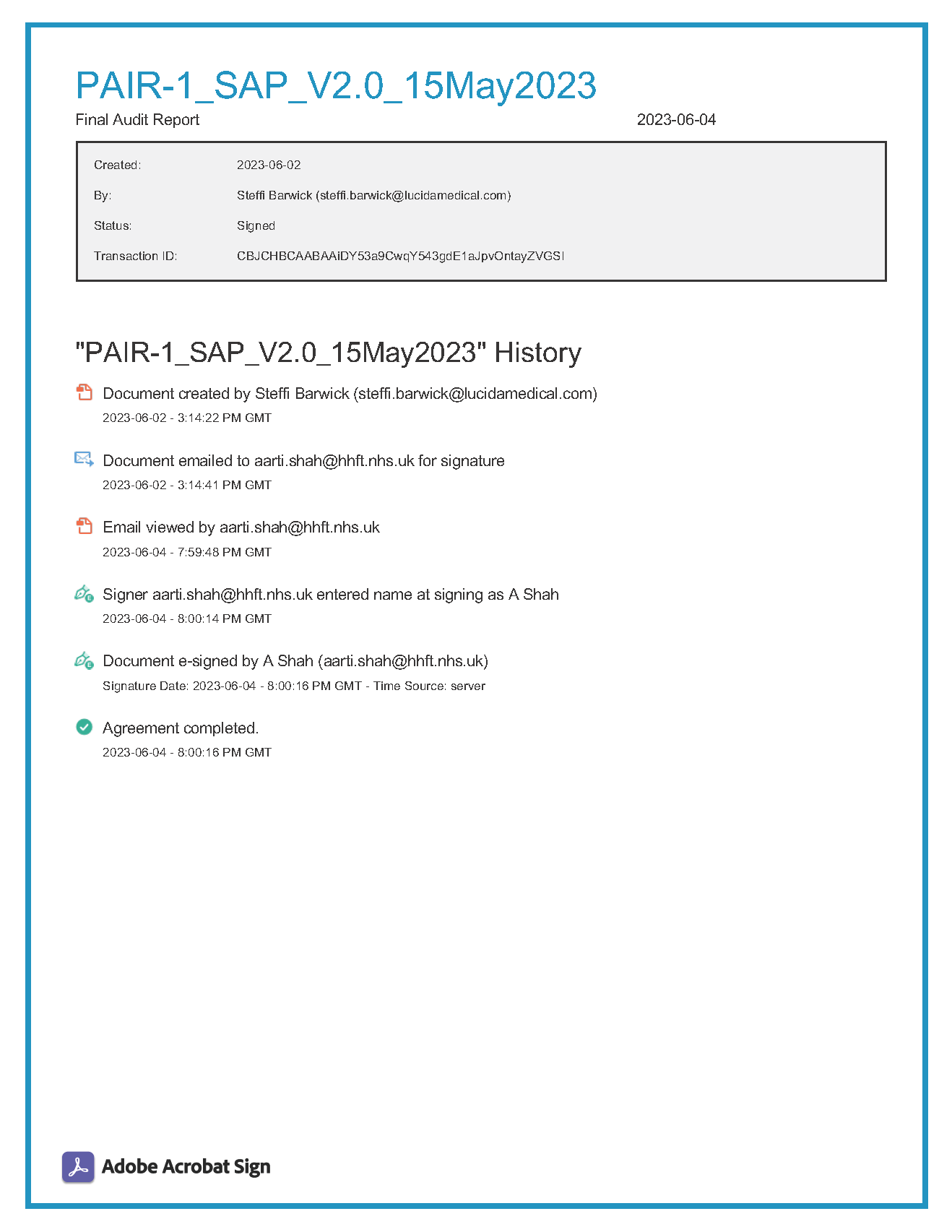
**

**Supplementary Material S4: Effect of scanner age and field strength**

Overall, per-patient sensitivity, specificity and PPV along with the number of patients stratified by magnetic field strength (1.5T vs 3T) and scanner age (< vs ≥ 8 years) are shown for multiparametric AI in the Table S2. The AI performed well (AUC ≥ 0.89) in all subgroups, with only small differences in AUC between subgroups.

|  |  | Patients | AUC | Sensitivity | Specificity | PPV |
| --- | --- | --- | --- | --- | --- | --- |
| Field Strength | 1.5T | 116 | 0.91(0.85-0.96) | 0.98 (0.91-1.00) | 0.61 (0.51-0.72) | 0.58 (0.46-0.69) |
|  | 3T | 136 | 0.90(0.84-0.96) | 0.92(0.82-1.00) | 0.70 (0.61-0.80) | 0.55 (0.42-0.66) |
| Scanner age | < 8 years | 126 | 0.92(0.86-0.97) | 0.97 (0.91-1.00) | 0.58 (0.48-0.68) | 0.47(0.36-0.58) |
|  | ≥ 8 years | 126 | 0.93(0.87-0.97) | 0.93 (0.85-1.00) | 0.76 (0.66-0.84) | 0.67(0.55-0.79) |

**Supplementary Material S4 Table** - Multiparametric AI performance on MRI stratified by field strength and scanner age. The AI performed well (AUC ≥ 0.89) in all subgroups, with only small differences in AUC between subgroups.

**Supplementary Material S5: AI false negatives**

As an exploratory endpoint, AI false negative lesions are reported in Table S3 at threshold 3.5. Most AI false negative lesions were GG2 (11/13 lesions) and 2 were GG3. For radiologists, there were 7 false negative lesions at the threshold of PI-RADS ≥ 3, comprising 5 cases of GG2 lesions, 1 case of GG3 and 1 case of GG4.

| Site (mpMRI) | Total Missed | GGG 2 | GGG 3 | GGG 4 | GGG 5 | PIRADS 0 | PIRADS 1 | PIRADS 2 | PIRADS 3 | PIRADS 4 | PIRADS 5 |
| --- | --- | --- | --- | --- | --- | --- | --- | --- | --- | --- | --- |
| 1 | 0 | 0 | 0 | 0 | 0 | 0 | 0 | 0 | 0 | 0 | 0 |
| 2 | 0 | 0 | 0 | 0 | 0 | 0 | 0 | 0 | 0 | 0 | 0 |
| 3 | 5 | 5 | 0 | 0 | 0 | 1 | 0 | 0 | 0 | 4 | 0 |
| 4 | 1 | 0 | 1 | 0 | 0 | 0 | 0 | 0 | 0 | 1 | 0 |
| 5 | 5 | 4 | 1 | 0 | 0 | 2 | 0 | 0 | 0 | 1 | 2 |
| 6 | 2 | 2 | 0 | 0 | 0 | 0 | 0 | 0 | 1 | 1 | 0 |

**Supplementary Material S5 Table** - False negative lesions by the multiparametric AI model separated by site and their Gleason Grade Group (GGG) and PI-RADS scores. PI-RADS 0 refers to visible and biopsy-confirmed lesions which were missed by the radiologists. 84.6% of the false negative lesions were GGG2.

**Supplementary Data**

*A set of figures and tables supporting the results on the main text:*


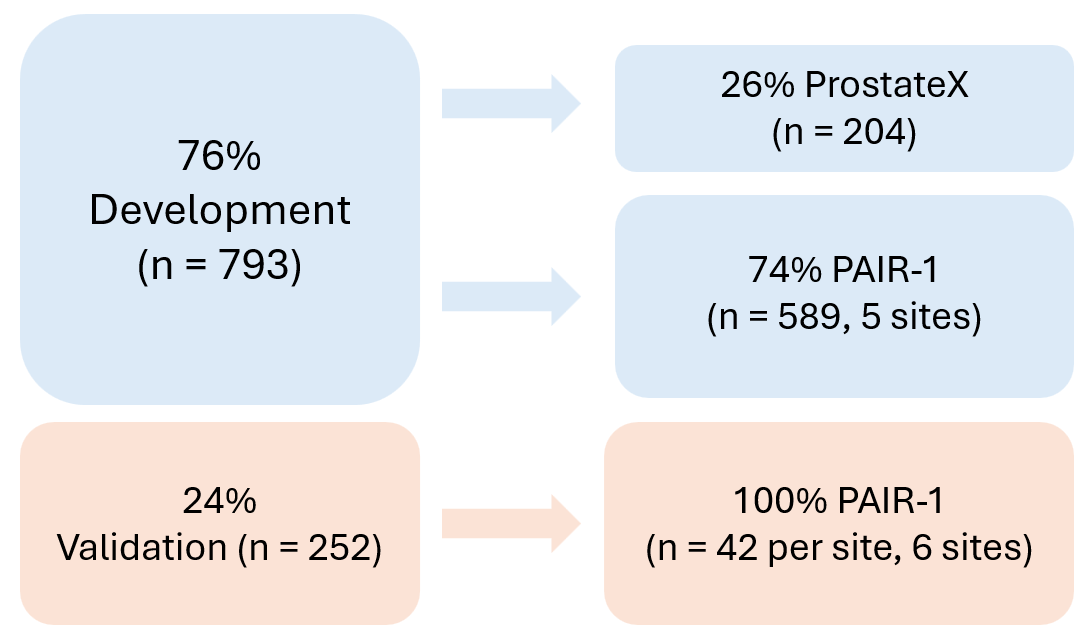


**Supplementary Figure 1** - Diagram of data partition for model development and validation.


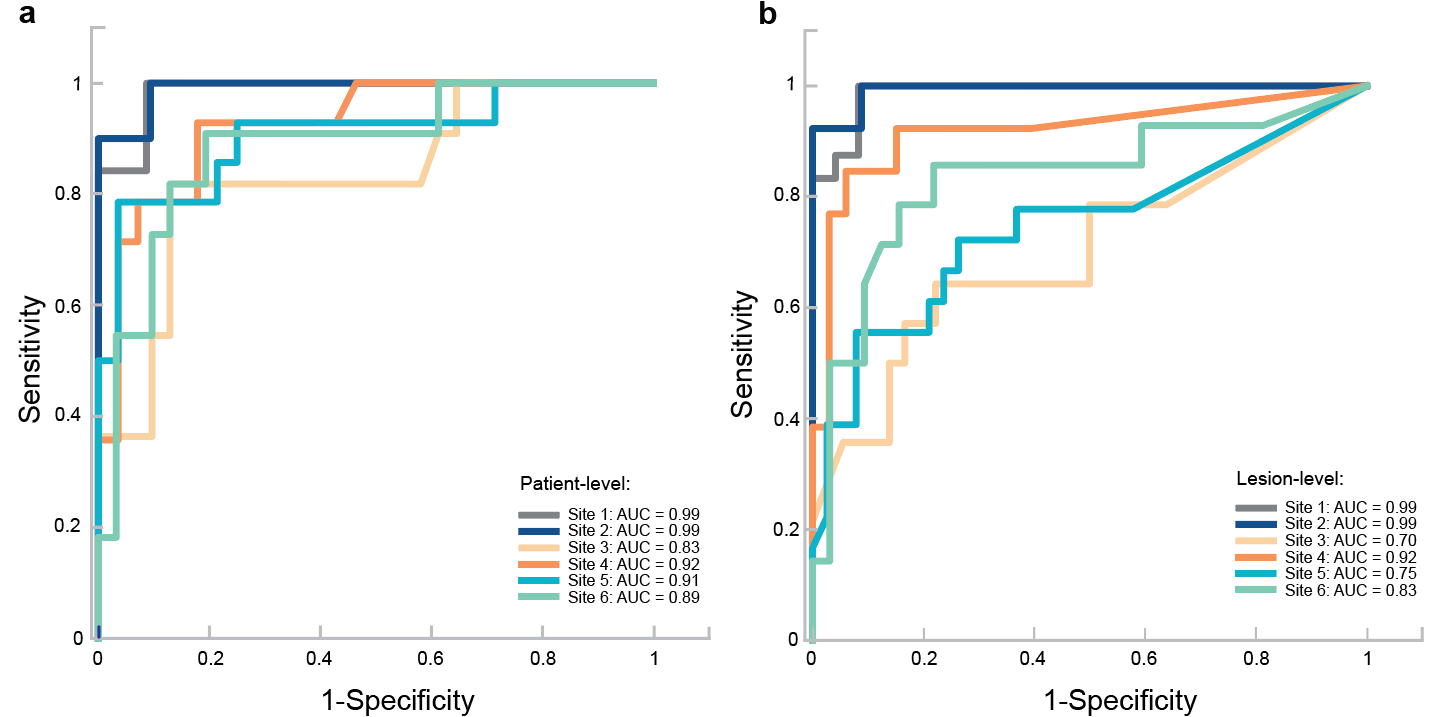


**Supplementary Figure 2** - (a) ROC curves at the patient level and (b) -lesion level for identifying csPCa, stratified by site with multiparametric AI model. The AUC values at patient level are consistently high across different sites, ranging from 0.83 to 0.99. The AUC for the site unseen during development (Site 4) was 0.92. At the lesion level, there is a broader range of AUC values, with two sites showing relatively low AUCs. Despite this, the AUC for the unseen site remained high at the lesion level, with a value of 0.92.

**Supplementary Table 1** - Number of patients and MR data for each centre.

|  | Number of patients  (Validation set) | Number of patients  (Development set) | Prevalence of GG>2 (Validation set) | MR scanner | Magnet | Scanner Age (years) | Multiparametric MR protocol |
| --- | --- | --- | --- | --- | --- | --- | --- |
| Site 1 | 42 | 93 | 45% | SIEMENS | 1.5 | 10 | Axial T2-WI, Sagittal T2-WI, DWI and DCE |
| Site 2 | 42 | 116 | 24% | SIEMENS | 3T | 3 | Axial T2-WI, Sagittal T2-WI, DWI and DCE |
| Site 3 | 42 | 128 | 26% | SIEMENS | 3T | 10 | Axial T2-WI, Sagittal T2-WI, DWI and DCE |
| Site 4 | 42 | 0 | 33% | SIEMENS | 1.5T | 3 | Axial T2-WI, Sagittal T2-WI, DWI and DCE |
| Site 5 | 42 | 134 | 33% | GE | 3T | 15 | Axial T2-WI, Sagittal T2-WI, DWI and DCE |
| Site 6 | 42 | 118 | 26% | SIEMENS | 1.5T (n=30)  3T (n=12) | 6  4 | Axial T2-WI, Sagittal T2-WI, DWI and DCE |

Legend – MR: Magnetic Resonance; GE: General Electric; T: Tesla; T2-WI: T2-weighted imaging; DWI: diffusion-weighted imaging; DCE: dynamic contrast enhanced

**Supplementary Table 2** – Demographic characteristics for the PAIR-1 development set used for model training.

| Development set (PAIR-1) | | csPCa | non-csPCa |
| --- | --- | --- | --- |
| Total (N) | | 203 | 386 |
| Age mean ± std | | 69.3±7.2 | 65.8±8.6 |
| PSA (ng/mL) median (IQR) | | 8.8 (IQR: 6.1 – 15.4)^*1^ | 5.7 (IQR: 4.2-8.4)^*2^ |
| BPH (N) | | 9^*3^ | 45^*4^ |
| Prostatitis (N) | | 3^*5^ | 16^*6^ |
| UTI (N) | | 7^*7^ | 33^*8^ |
| Family history  Genetic predisposition (N) | | 24^*9^ | 49^*10^ |
| LUTS (N) | | 109^*11^ | 207^*12^ |
| Prostate Volume (mL) mean ± std | | 50.8±25.8 | 73.5±51.7 |
| PI-RADS (N) | 1  2  3  4  5 | 1  1  7  61  134 | 1  263  68  32  22 |
| Gleason Grade Group (N) | Negative  Not available  1  2  3  4  5 | 0  0  0  99  39  21  44 | 106  238  42  0  0  0  0 |

Missing data: ^*1^ - 1 patient without PSA information, ^*2^  - 2 patients without PSA information, ^*3^ - 61 patients without BPH information, ^*4^ - 90 patients without BPH information, ^*5^ - 62 patients without Prostatitis information, ^*6^ - 93 patients without Prostatitis information, ^*7^ - 58 patients without UTI information, ^*8^ - 90 patients without UTI information, ^*9^ - 65 patients without FH/GP information, ^*10^ - 114 patients without FH/GP information, ^*11^ - 55 patients without LUTS information, ^*12^ - 83 patients without LUTS information

**Supplementary Table 3** – Demographics characteristics for the validation set (N = 252).

| **Validation set** | | **csPCa** | **non-csPCa** |
| --- | --- | --- | --- |
| **Total (N)** | | 79 | 173 |
| **Age mean ± std** | | 69.4±7.8 | 65.8±8.6 |
| **PSA (ng/mL) median (IQR)** | | 10.2 (IQR: 6.3 - 14.5) | 5.9 (IQR: 4.1-8.8) |
| **BPH (N)** | | 1^*1^ | 16^*2^ |
| **Prostatitis (N)** | | 0^*3^ | 3^*4^ |
| **UTI (N)** | | 2^*5^ | 10^*6^ |
| **Family history**  **Genetic predisposition (N)** | | 11^*7^ | 24^*8^ |
| **LUTS (N)** | | 44^*9^ | 91^*10^ |
| **Prostate Volume (mL) mean ± std** | | 50.2±26.9 | 71.5±39.0 |
| **PI-RADS (N)** | 1  2  3  4  5 | 0  1  4  22  52 | 4  123  23  17  6 |
| **Gleason Grade Group (N)** | Negative  Not available  1  2  3  4  5 | 0  0  0  41  18  5  15 | 41  115  17  0  0  0  0 |

Missing data: ^*1^ - 26 patients without BPH information, ^*2^ - 57 patients without BPH information, ^*3^ - 26 patients without Prostatitis information, ^*4^ - 62 patients without Prostatitis information, ^*5^ - 26 patients without UTI information, ^*6^ - 62 patients without UTI information, ^*7^ - 21 patients without FH/GP information, ^*8^ - 55 patients without FH/GP information, ^*9^ - 15 patients without LUTS information, ^*10^ - 50 patients without LUTS information

**Supplementary Table 4** – Demographics characteristics per site.

| **Demographics** | | **Validation** | | | | | | **Development** | | | | |
| --- | --- | --- | --- | --- | --- | --- | --- | --- | --- | --- | --- | --- |
|  |  | **Site 1** | **Site 2** | **Site 3** | **Site 4** | **Site 5** | **Site 6** | **Site 1** | **Site 2** | **Site 3** | **Site 5** | **Site 6** |
| **Age** **mean ± std** | | 70.6±7.1 | 66.2±9.3 | 63.0±5.9 | 70.3±7.3 | 65.8±9.7 | 65.8±9.2 | 68.2±8.2 | 68.8±8.4 | 65.7±8.5 | 65.9±8.1 | 66.8±8.1 |
| **Prostate Volume (mL) mean ± std** | | 72.0±36.7 | 59.1±40.6 | 59.2±20.6 | 73.9±42.3 | 65.4±38.4 | 59.3±38.5 | 66.1±40.7 | 66.2±46.6 | 60.9±34.2 | 72.2±64.4 | 62.4±32.6 |
| **PSA level** (**ng/mL) median (IQR** | | 9.0 (IQR: 5.7 – 13.5) | 6.0 (IQR: 2.9 – 8.4) | 5.5 (IQR: 4.1 – 7.1) | 9.4 (IQR: 6.4 – 15.1) | 6.3 (IQR: 4.6 – 9.4) | 6.6 (IQR: 4.4 – 10.0) | 6.4 (IQR: 4.4 – 8.8) | 8.2 (IQR: 5.34 – 11.1) | 6.1 (IQR: 4.6 – 10.4) | 6.0 (IQR: 4.4 – 9.4) | 6.6 (IQR: 4.7 – 9.7) |
| **PI-RADS (N)** | 1 | 0 | 0 | 0 | 0 | 0 | 4 | 0 | 0 | 0 | 0 | 1 |
|  | 2 | 21 | 22 | 21 | 21 | 20 | 19 | 38 | 50 | 56 | 69 | 51 |
|  | 3 | 2 | 5 | 11 | 1 | 3 | 5 | 18 | 13 | 18 | 12 | 13 |
|  | 4 | 4 | 3 | 6 | 11 | 7 | 8 | 17 | 10 | 23 | 20 | 24 |
|  | 5 | 15 | 12 | 4 | 9 | 12 | 6 | 20 | 43 | 31 | 33 | 29 |
| **Gleason Grade Group (N)** | Negative | 1 | 7 | 13 | 4 | 9 | 7 | 12 | 23 | 29 | 20 | 22 |
|  | Not available | 21 | 18 | 17 | 19 | 18 | 22 | 47 | 40 | 46 | 63 | 42 |
|  | 1 | 1 | 7 | 1 | 5 | 1 | 2 | 6 | 4 | 12 | 10 | 10 |
|  | 2 | 8 | 2 | 7 | 8 | 9 | 7 | 10 | 26 | 22 | 16 | 25 |
|  | 3 | 6 | 3 | 2 | 3 | 3 | 1 | 8 | 7 | 9 | 7 | 8 |
|  | 4 | 1 | 0 | 2 | 1 | 0 | 1 | 7 | 4 | 7 | 0 | 3 |
|  | 5 | 4 | 5 | 0 | 2 | 2 | 2 | 3 | 12 | 3 | 16 | 8 |

**Supplementary Table 5** - Average number of false positives per patient from fROC analysis at specific thresholds for multiparametric AI model and PIRADS, showing a consistent lower number of false positives for the expert radiologist.

|  | Sensitivity | Average number of false positives per patient |
| --- | --- | --- |
| mpMRI AI | 0.8 | 0.31 |
|  | 0.9 | 1.54 |
| Radiologists | 0.8 | 0.12 |
|  | 0.9 | 0.21 |

Legend: mpMRI: multiparametric magnetic resonance imaging; AI: artificial intelligence

**Supplementary Table 6** - Lesion ROC analysis, showing the overall sensitivity, specificity and PPV for AI model and expert radiologists at specified thresholds of 3.5 and 3, respectively. This table also includes performance for the AI model stratified per site. Only lesions identified by the original radiologists or through systematic biopsy are considered, reflecting the use of AI as a confirmatory tool or second reader. Overall, the multiparametric AI model demonstrates performance comparable to that of radiologists across most sites. However, variability between sites, underscore the need for site-specific adjustments on the model threshold to improve models’ accuracy, in particular for the sites 3, 5 and 6.

| **Lesion level** | | **Sensitivity** | **Specificity** | **PPV** | **AUC** |
| --- | --- | --- | --- | --- | --- |
| **Overall** | PI | 0.87 (0.79-0.93) | 0.73 (0.66-0.79) | 0.61 (0.53-0.68) | 0.86 (0.81-0.91) |
|  | Radiologists | 0.93 (0.87-0.98) | 0.69 (0.62-0.75) | 0.59 (0.51-0.67) | 0.86 (0.80-0.91) |
|  | Site 1 | 1.00 (1.00-1.00) | 0.83 (0.68-0.96) | 0.86 (0.72-0.97) | 0.99 (0.96-1.00) |
|  | Site 2 | 1.00 (1.00-1.00) | 0.79 (0.65-0.92) | 0.65 (0.44-0.86) | 0.99 (0.97-1.00) |
|  | Site 3 | 0.64 (0.36-0.89) | 0.78 (0.63-0.91) | 0.53 (0.27-0.78) | 0.70 (0.52-0.88) |
|  | Site 4 | 0.92 (0.75-1.00) | 0.85 (0.72-0.96) | 0.71 (0.47-0.92) | 0.92 (0.80-1.00) |
|  | Site 5 | 0.72 (0.50-0.92) | 0.71 (0.56-0.85) | 0.54 (0.35-0.74) | 0.75 (0.58-0.89) |
|  | Site 6 | 0.86 (0.64-1.00) | 0.41 (0.24-0.57) | 0.39 (0.21-0.57) | 0.83 (0.66-0.97) |
